# Chemical engineering of γδ T cells with cancer cell-targeting antibodies for enhanced tumor immunotherapy

**DOI:** 10.1093/nsr/nwaf256

**Published:** 2025-06-27

**Authors:** Long Chen, Bo Cheng, Zhanqun Yang, Mengzhu Zheng, Tianyu Chu, Pan Wang, Tianhui He, Yuan Xue, Houyi Ren, Liting Zheng, Peng Zhou, Xiaxuan Li, Haichuan Zhu, Hongyan Guo, Xing Chen, Jian Lin

**Affiliations:** Department of Pharmacy, Peking University Third Hospital Cancer Center, Peking University Third Hospital, Beijing 100191, China; College of Chemistry and Molecular Engineering, Peking University, Beijing 100871, China; Beijing National Laboratory for Molecular Sciences, Peking University, Beijing 100871, China; School of Pharmaceutical Sciences, Peking University, Beijing 100191, China; Department of Pharmacy, Peking University Third Hospital Cancer Center, Peking University Third Hospital, Beijing 100191, China; Key Laboratory of Tropical Biological Resources of Ministry of Education, Song Li's Academician Workstation of Hainan University, School of Pharmaceutical Sciences, Hainan University, Haikou 572000, China; College of Chemistry and Molecular Engineering, Peking University, Beijing 100871, China; Beijing National Laboratory for Molecular Sciences, Peking University, Beijing 100871, China; Department of Obstetrics and Gynecology, Peking University Third Hospital, Beijing 100191, China; Department of Obstetrics and Gynecology, Peking University Third Hospital, Beijing 100191, China; Department of Pharmacy, Peking University Third Hospital Cancer Center, Peking University Third Hospital, Beijing 100191, China; Department of Pharmacy, Peking University Third Hospital Cancer Center, Peking University Third Hospital, Beijing 100191, China; Artificial Auditory Laboratory of Jiangsu Province, Xuzhou Medical University, Xuzhou 221004, China; LinXCell Biotechnologies, Beijing 102600, China; Key Laboratory of Tropical Biological Resources of Ministry of Education, Song Li's Academician Workstation of Hainan University, School of Pharmaceutical Sciences, Hainan University, Haikou 572000, China; School of Life Science and Health, Wuhan University of Science and Technology, Wuhan 430081, China; Department of Obstetrics and Gynecology, Peking University Third Hospital, Beijing 100191, China; College of Chemistry and Molecular Engineering, Peking University, Beijing 100871, China; Beijing National Laboratory for Molecular Sciences, Peking University, Beijing 100871, China; Peking-Tsinghua Center for Life Sciences, Peking University, Beijing 100871, China; Synthetic and Functional Biomolecules Center, Peking University, Beijing 100871, China; Key Laboratory of Bioorganic Chemistry and Molecular Engineering of Ministry of Education, Peking University, Beijing 100871, China; Department of Pharmacy, Peking University Third Hospital Cancer Center, Peking University Third Hospital, Beijing 100191, China; Key Laboratory of Tropical Biological Resources of Ministry of Education, Song Li's Academician Workstation of Hainan University, School of Pharmaceutical Sciences, Hainan University, Haikou 572000, China; Synthetic and Functional Biomolecules Center, Peking University, Beijing 100871, China; College of Life Science, Anhui Medical University, Hefei 230032, China

**Keywords:** chemical engineering, metabolic glycan labeling, sialic acid, antibody-γδ T cell conjugate, tumor microenvironment

## Abstract

Gamma delta (γδ) T cells hold great promise in adoptive cell therapy, but suffer from low tumor-targeting efficiency. Herein, we report the development of antibody-γδ T cell conjugates for enhanced tumor therapy. By evaluating different biomolecules residing on the cell surface, sialic acids—the terminal sugars of various cell-surface glycans—are identified as the optimum site for anchoring antibodies onto γδ T cells via metabolic glycan labeling with unnatural sugars containing a bioorthogonal functional group. A programmed death-ligand 1 (PD-L1)-specific nanobody (αPD-L1) is conjugated onto γδ T cells via click chemistry and the resulting αPD-L1-γδ T cells exhibit enhanced cytotoxicity towards PD-L1-positive cancer cell lines, patient-derived primary cancer cells, and xenografted tumors in living mice. Mechanistically, αPD-L1-γδ T cells target cancer cells and tumors via binding to PD-L1 and induce cancer cell pyroptosis. Furthermore, αPD-L1-γδ T cells remodel the tumor microenvironment to be immune-active, at least partially through the recruitment and activation of CD8^+^ T cells via the CCR5/CCL5 axis. This work provides a versatile strategy for chemical engineering of γδ T cells for improved therapeutic applications.

## INTRODUCTION

In contrast to the conventional alpha beta (αβ) T cell, gamma delta (γδ) T cells are a unique subset of T lymphocytes that possess distinct T cell receptors (TCRs), i.e. gamma and delta chains [1,2]. Unlike the major histocompatibility complex (MHC)-dependent antigen recognition by αβ TCRs, TCRs of γδ T cells directly recognize the protein antigen, the BTN3A1/BTN2A1 complex formed with phosphoantigens as molecular glues [3–5]. The MHC-independent mechanism for antigen recognition makes γδ T cells an ideal choice for allogeneic cell therapy because no graft-versus-host disease would be expected. γδ T cells have been shown to exert anti-tumor activity both *in vitro* and *in vivo*, attracting extensive interests in the development of adoptive cell therapy based on γδ T cells [1,6–8]. However, like most immune cells used for adoptive cell transfer, *ex vivo* expanded and unengineered γδ T cells usually result in limited clinical benefits due to low tumor-targeting efficiency and limited activation of the transferred cells [9–11].

To overcome these limitations, several engineering approaches have been developed to elevate the anti-tumor efficacy of γδ T cells. For example, chimeric antigen receptors (CARs) have been engineered to γδ T cells, inspired by the great success of αβ CAR-T cells [10–13]. Albeit promising, genetic engineering and large-scale production of γδ CAR-T cells remain costly and time-consuming [14].

Alternatively, chemical engineering of cell surfaces represents a versatile and promising way to modulate the functions of immune cells such as natural killer cells and αβ CAR-T cells [15–18]. We therefore envisioned that chemical engineering of the γδ T cell surface with a tumor-targeting functionality would provide engineered γδ T cells with enhanced anti-tumor activity and ease of production.

As to the possible anchoring sites on the cell surface, there are various kinds of biomolecules, such as lipids, proteins and glycans, with distinct functions and varied distances from the cell membrane [19]. We rationalized that chemical engineering on different cell-surface components may result in different targeting efficacies and anti-tumor activities. On the other hand, various chemical engineering strategies have been developed for different biomolecules. Metabolic glycan labeling (MGL) exploits the biosynthetic pathways of cellular glycans to incorporate unnatural monosaccharides containing a bioorthogonal functional group such as azide, which can then be functionalized via bioorthogonal chemistry or click chemistry [20–23]. A list of unnatural monosaccharides is currently available for labeling specific glycans [23], which in principle provides different engineering sites within the glycan chains. Similarly, lipid analogs and noncanonical amino acids containing a bioorthogonal functional group have also been developed for metabolic labeling of lipids [24] and proteins [25], respectively.

Based on these considerations, we herein report the development of antibody–γδ T cell conjugates by chemical engineering of cell-surface glycans, and demonstrate the enhanced anti-tumor efficacy both *in vitro* and *in vivo*. By evaluating different cell-surface biomolecules, cell-surface sialic acids, the terminal sugars of various cell-surface glycans, were chosen as the anchoring site for tumor-targeting antibodies. By exploiting the fast MGL (fMGL) strategy recently developed based on 1-*O*-acetyl-*N*-azidoacetyl-mannosamine-6-bis(*S*-acetyl-2-thioethyl)-phosphate (AMS-ManNAz-P) [26], the antibodies were efficiently and rapidly conjugated onto cell-surface sialoglycans with no interference with γδ T cell functions. The γδ T cells conjugated with an anti-PD-L1 antibody (αPD-L1-γδ T cells) exhibited efficient killing towards PD-L1-positive cancer cell lines and patient-derived primary cancer cells, and inhibited the tumor growth in xenograft mouse models. Mechanistically, the binding of PD-L1 by αPD-L1 targeted αPD-L1-γδ T cells to cancer cells and tumors, which induced pyroptosis of cancer cells. Furthermore, infiltration of αPD-L1-γδ T cells shaped the immunoactive tumor microenvironment (TME), at least partially through the recruitment and activation of CD8^+^ T cells via the CCR5/CCL5 axis. The antibody–γδ T cell conjugates developed in this work thus provide a promising strategy for engineering γδ T cells with enhanced anti-tumor activity.

## RESULTS

### Conjugation of antibodies on cell-surface sialic acids of γδ T cells

**Figure 1.**
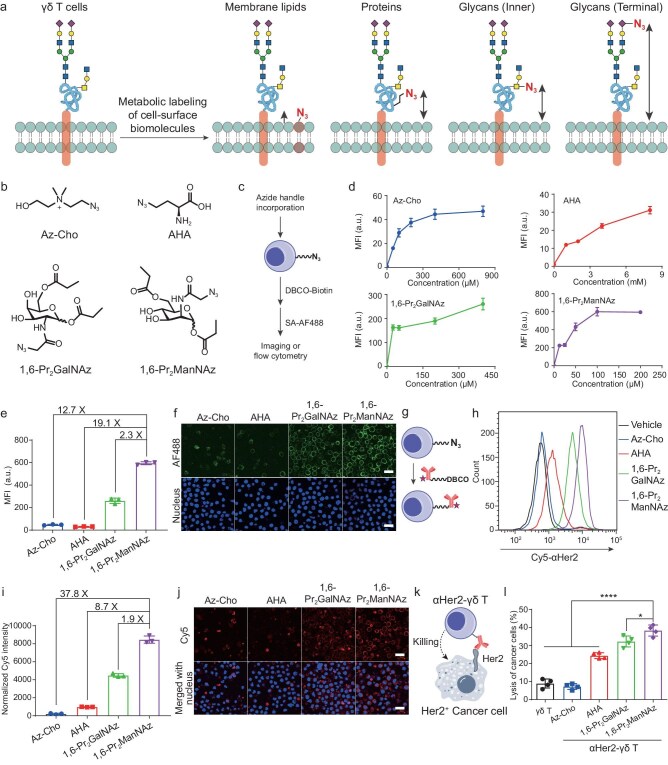
Conjugation of antibodies onto the surface of γδ T cells. (a) Schematic showing the metabolic labeling strategy for installation of azides onto membrane lipids, cell-surface proteins, the inner part of *O*-glycans and *N*-glycans, and terminal sialic acid residues, respectively. (b) Structures of chemical reporters including Az-Cho, AHA, 1,6-Pr_2_GalNAz and 1,6-Pr_2_ManNAz. (c) Schematic of click labeling of azide-incorporated γδ T cells with fluorophores. (d) Flow cytometry analysis showing the concentration-dependent incorporation of azides in γδ T cells, which were incubated with Az-Cho, AHA, 1,6-Pr_2_GalNAz, or 1,6-Pr_2_ManNAz at varied concentrations, followed by reaction with DBCO-biotin and staining with streptavidin-AF488. (e, f) Flow cytometry analysis (e) and confocal fluorescence microscopy images (f) of γδ T cells incubated with 800 μM Az-Cho, 8 mM AHA, 400 μM 1,6-Pr_2_GalNAz or 200 μM 1,6-Pr_2_ManNAz, followed by click labeling and flow cytometry analysis. In (e), the data were from (d). (g) Schematic showing conjugation of antibodies via click chemistry onto γδ T cells. Red pentagram indicates labeled Cy5 fluorophore on antibody. (h–j) Flow cytometry analysis (h, representative histogram; i, bar graph) and confocal fluorescence microscopy images (j) of γδ T cells incubated with 100 μM Az-Cho, 8 mM AHA, 400 μM 1,6-Pr_2_GalNAz or 200 μM 1,6-Pr_2_ManNAz, followed by reaction with Cy5-conjugated αHER2-DBCO. (k) Schematic showing αHer2-γδ T cells killing Her2^+^ cancer cells. (l) Percentages of killed MDA-MB-231-hHER2 cells after incubation with γδ T cells or αHer2-γδ T cells at the effector-to-target ratio of 1:1 for 3 h. The αHer2-γδ T cells were prepared by labeling with 100 μM Az-Cho, 8 mM AHA, 400 μM 1,6-Pr_2_GalNAz or 200 μM 1,6-Pr_2_ManNAz. * *P* < 0.05, **** *P* < 0.0001 (one-way ANOVA). In (d), (e), (i) and (l), data are presented as mean ± SD [*n *= 4 in (l) and 3 in the rest]. In (f) and (j), scale bars are 20 μm.

We started out by evaluating different sites on the γδ T cell surface for conjugation of targeting antibodies (Fig. 1a). Human γδ T cells were expanded from peripheral blood mononuclear cells (PBMCs) with >95% purity using a previously reported protocol [27] with slight modifications (Fig. S1a). Azido-choline [24] (Az-Cho), *L*-azidohomoalanine [25] (AHA), 1,6-di-*O*-propionyl-*N*-azidoacetylgalactosamine (1,6-Pr_2_GalNAz) [28,29] and 1,6-di-*O*-propionyl-*N*-azidoacetylmannosamine (1,6-Pr_2_ManNAz) [29] were employed for metabolically installing azides as the bioorthogonal handle onto membrane lipids, cell-surface proteins, the inner part of mucin-type *O*-glycans and *N*-glycans, and the terminal sialic acid moiety of sialoglycans, respectively, which reside with increasing distances from the membrane (Fig. 1a and b). The γδ T cells were incubated with Az-Cho, AHA, 1,6-Pr_2_GalNAz or 1,6-Pr_2_ManNAz at varied concentrations for 24 h, reacted with dibenzocyclooctyne (DBCO)-biotin via copper-free click chemistry, and stained with streptavidin-Alexa Fluor 488 (AF488), followed by analysis with confocal fluorescence microscopy and flow cytometry (Fig. 1c). Concentration-dependent cell-surface labeling was observed for all azido analogs (Fig. 1d). No apparent cell cytotoxicity was observed at any of the concentrations tested (Fig. S1b). Labeling by 1,6-Pr_2_ManNAz reached saturation at the concentration of 100 μM, while the Az-Cho at 800 μM, AHA at 8 mM and 1,6-Pr_2_GalNAz at 400 μM did not result in saturated labeling (Fig. 1d). Notably, the labeling intensity of 1,6-Pr_2_ManNAz at 100 μM was much higher than the highest labeling intensities by the other three azido analogs (Fig. 1e and f).

Not only should the 1,6-Pr_2_ManNAz labeling result in the most attachment sites, but also the outermost position of azido sialic acid (SiaNAz) [29] would endow the best accessibility for antibodies. To evaluate this hypothesis, anti-HER2 IgG monoclonal antibody (αHER2) was functionalized with DBCO by the reaction of the lysine residues with DBCO-*N*-hydroxysuccinimidyl (DBCO-NHS) ester (Fig. S1c). The resulting αHER2-DBCO then reacted with human γδ T cells incubated with the azido analogs (Fig. 1g). Flow cytometry and confocal fluorescence microscopy analysis showed that 1,6-Pr_2_ManNAz labeling produced the highest amount of αHER2 on the cell surface (Fig. 1h–j). Notably, the anti-HER2 antibody conjugated to sialic acids could also be more easily recognized by the HER2 receptor on cancer cells owing to less steric hindrance.

MDA-MB-231-hHER2 cells, a HER2-overexpressing breast cancer line, were incubated with γδ T cells conjugated with αHER2, referred to as αHER2-γδ T cells hereafter (Fig. 1k). The αHER2-γδ T cells based on 1,6-Pr_2_ManNAz labeling exhibited the highest cytotoxicity and cancer cell-killing efficiency (Fig. 1l). The advantage of 1,6-Pr_2_ManNAz over 1,6-Pr_2_GalNAz was further confirmed in another HER2^+^ breast cancer cell line, MDA-MB-453 cells (Fig. S1d and e). These results demonstrate that by engineering tumor cell-targeting antibodies at the outermost layer of cell surfaces, i.e. cell-surface sialic acids, the antibody-γδ T cell conjugates are endowed with improved cancer cell-killing efficacy.

### Fast and specific labeling of sialic acids on γδ T cell surface with AMS-ManNAz-P

**Figure 2.**
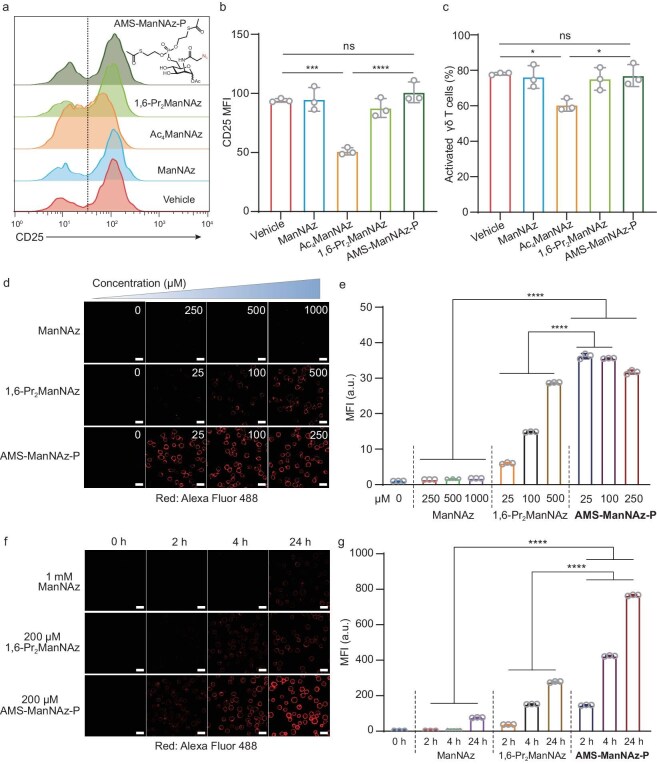
Fast and specific labeling of cell-surface sialic acids with AMS-ManNAz-P in γδ T cells. (a–c) Representative histogram (a), bar graph of flow cytometry (b) and percentages of activation (c) of γδ T cells incubated with vehicle, 1 mM ManNAz, 200 μM Ac_4_ManNAz, 200 μM 1,6-Pr_2_ManNAz or 200 μM AMS-ManNAz-P for 24 h, followed by staining of an anti-CD25 antibody. (d, e) Confocal fluorescence microscopy images (d) and quantitative flow cytometry analysis (e) of the dose-dependent cell surface engineering efficacy using different unnatural monosaccharides after 48 h incubation. AMS-ManNAz-P showed the highest MGL efficacy. (f, g) Confocal fluorescence microscopy images (f) and quantitative flow cytometry analysis (g) of the time-dependent cell surface engineer using different unnatural monosaccharides. AMS-ManNAz-P showed the fastest MGL kinetics and highest MGL efficacy. Data are presented as mean ± SD. *n* = 3. ns, not significant, *P < 0.05, ***P < 0.001, ****P < 0.0001 (one-way ANOVA). In (d) and (f), scale bars are 20 μm.

After choosing cell-surface sialic acids as the conjugation sites, we optimized the labeling protocol. In addition to 1,6-Pr_2_ManNAz, AMS-ManNAz-P (Fig. 2a and Fig. S2a) was recently developed as an unnatural sugar with a faster labeling rate [26], which should be beneficial for constructing antibody–γδ T cell conjugates. The former generations of unnatural sugars including *N*-azidoacetylmannosamine (ManNAz) and per-*O*-acetylated-N-azidoacetylmannosamine (Ac_4_ManNAz) were also included for comparison (Fig. S2a).

Per-*O*-acetylated unnatural sugars, including Ac_4_ManNAz, can induce non-specific *S*-glyco-modifications on cysteine residues of various proteins [30,31] (Fig. S2a). Since *S*-glyco-modification is non-enzymatic, direct incubation of unnatural sugars with cell lysates can be used to assay *S*-glyco-modification. In the lysates of γδ T cells, Ac_4_ManNAz, but not AMS-ManNAz-P, induced protein *S*-glyco-modification (Fig. S2b). To evaluate possible effects of *S*-glyco-modification on γδ T cell functions, we performed bioinformatics analysis on the previously identified dataset of *S*-glyco-modified proteins [32]. Kyoto Encyclopedia of Genes and Genomes (KEGG) and Gene Ontology (GO) analysis showed that the modified proteins were enriched in signaling pathways related to T cell functions (Fig. S2c) and molecular functions related to TCR signaling, T cell activation and natural killer (NK) cell activation (Fig. S2d). RhoA, a GTPase universally expressed in various T cell types (Fig. S2e) and essential for T cell functions [33], was among the *S*-glyco-modified proteins. Two out of the three modified cysteine residues were in close proximity with the GTP binding site (Fig. S2f). *In vitro* enzymatic activity assay revealed that RhoA activity was reduced by Ac_4_ManNAz, but not by AMS-ManNAz-P (Fig. S2g). The effects of unnatural sugars on γδ T cell activation were then assayed by staining the activation marker CD25. Flow cytometry analysis showed that the γδ T cell activation was impaired by Ac_4_ManNAz, but not by AMS-ManNAz-P or other azido sugars not inducing *S*-glyco-modification (Fig. 2a–c). These results demonstrate that γδ T cells are sensitive to *S*-glyco-modification, which can inhibit γδ T cell activation.

To evaluate the labeling efficacy and labeling rate of unnatural sugars for MGL in γδ T cells, γδ T cells were incubated with ManNAz, 1,6-Pr_2_ManNAz or AMS-ManNAz-P for 48 h at varied concentrations, followed by reaction with DBCO-biotin and staining with streptavidin-AF488. Confocal microscopy and flow cytometry analysis exhibited cell-surface labeling in an AMS-ManNAz-P concentration-dependent manner (Fig. 2d and e). Of note, AMS-ManNAz-P at 25 μM resulted in a higher labeling intensity than other azido sugars at all concentrations tested. The γδ T cells were then incubated with 1 mM ManNAz, 200 μM 1,6-Pr_2_ManNAz or 200 μM AMS-ManNAz-P for varied durations of time. At 2 h, marked cell-surface labeling was observed for AMS-ManNAz-P, with a much higher signal-to-noise ratio than the other two azido sugars (Fig. 2f and g). Although the labeling intensity increased over time for all three azido sugars, AMS-ManNAz-P at 4 h surpassed ManNAz and 1,6-Pr_2_ManNAz at 24 h. To validate that AMS-ManNAz-P installed the azides on cell-surface sialic acids, the γδ T cells incubated with AMS-ManNAz-P were subjected to glycoproteomics analysis by click-iG [34], which elucidated the glycan compositions of azide-incorporated glycopeptides (Fig. S3a). For the identified glycopeptides including *N*-glycans and mucin-type *O*-glycans, the azides were mostly assigned to the terminal sialic acids (Fig. S3b and c). In addition, AMS-ManNAz-P did not induce cytotoxicity to γδ T cells (Fig. S3d). Together, these results demonstrate that AMS-ManNAz-P labels cell-surface sialic acids with fast labeling kinetics and high efficacy in γδ T cells, which should minimize perturbation to γδ T functions and exhaustion caused by prolonged *ex vivo* culture.

AMS-ManNAz-P-based fMGL followed by click reaction with DBCO-modified probes including small molecules, proteins and nuclear acids should enable functionalization of cell surfaces with various functionalities (Fig. S4a). As a proof-of-concept experiment, we first engineered γδ T cells with green fluorescent protein (GFP) via incubation with AMS-ManNAz-P and reaction with DBCO-GFP. Flow cytometry and confocal fluorescence microscopy analysis confirmed green fluorescence on the cell surface of γδ T cells (Fig. S4b and c). Similarly, by using a DBCO-modified DNA aptamer that targets PD-L1 [35], installation of nucleic acids onto the surface of γδ T cells was demonstrated (Fig. S4d and e). In the experiments using DBCO-biotin (Fig. 2d–g), AMS-ManNAz-P-based fMGL was shown to install small molecules onto the γδ T cells. Taken together, these results demonstrate that fMGL using AMS-ManNAz-P can efficiently engineer cells with diversified functional molecules, thus providing an optimized platform for γδ T cell engineering.

### αPD-L1-γδ T cells with enhanced cytotoxicity toward PD-L1-positive cell lines and primary cancer cells

**Figure 3.**
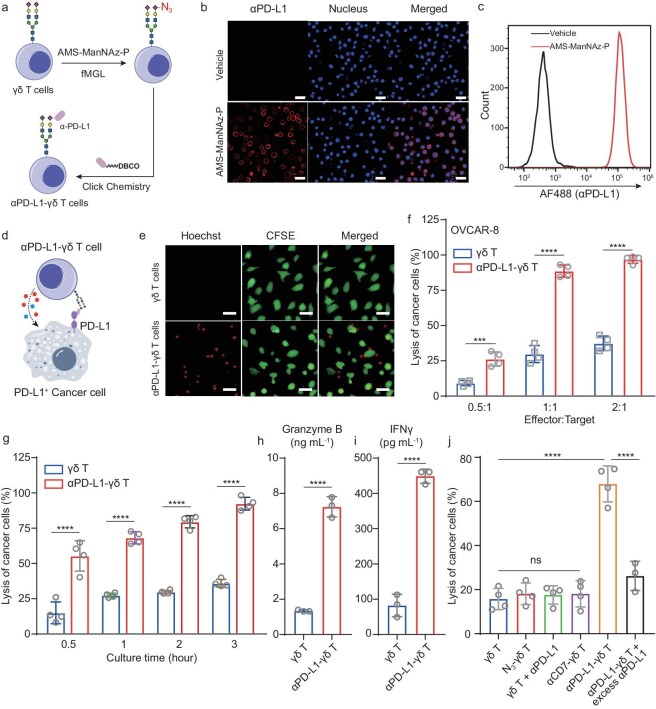
αPD-L1-γδ T cells with improved cytotoxicity. (a) Schematic of procedures for cell-surface engineering of γδ T cells with αPD-L1. (b, c) Confocal fluorescence microscopy images (b) and flow cytometry analysis (c) of αPD-L1-γδ T cells, which were stained with biotinylated PD-L1 (the extracellular domain) and streptavidin-AF488. (d) Schematic of αPD-L1-γδ T cells binding and killing PD-L1-positive cancer cells. (e) Confocal fluorescence microscopy images of γδ T cells or αPD-L1-γδ T cells incubated with OVCAR-8 cells. The γδ T and αPD-L1-γδ T cells were stained with Hoechst 33342 dye (red) and U251 cells with carboxyfluorescein succinimidyl ester (CFSE) (green). (f) *In vitro* cytotoxicity of γδ T and αPD-L1-γδ T cells against U251 cells at varied ratios of the effector to target cells. The percentages of lysed OVCAR-8 cells were quantified at 3 h of co-culture. (g) Time-course quantification of OVCAR-8 cells killed by γδ T cells or αPD-L1-γδ T cells at the effector to target cell ratio of 1:1. (h, i) Release of GrB (h) and IFNγ (i) in γδ T cells and αPD-L1-γδ T cells upon incubation with OVCAR-8 cells, which were assayed by ELISA. (j) Quantification of OVCAR-8 cells killed by γδ T cells, SiaNAz-incorporated γδ T cells, αPD-L1, mixture of αPD-L1 and γδ T cells, αCD7-γδ T cells, αPD-L1-γδ T cells or αPD-L1-γδ T cells together with excess αPD-L1. In (b) and (e), scale bars are 20 μm. In (f) and (g), the data are presented as mean ± SD [*n* = 3 for (h, i) and 4 for (f, g, j)]. ns, not significant, ***P < 0.001, ****P < 0.0001 [unpaired Student's *t*-test in (h, i) and one-way ANOVA in (f, g, j)].

To target γδ T cells to tumors by using AMS-ManNAz-P-based fMGL, we chose to install an anti-PD-L1 nanobody (αPD-L1) [36], given that PD-L1 is overexpressed in various tumors [37] (Fig. 3a). For functionalization of αPD-L1 with DBCO via lysine chemistry, the stoichiometry was controlled to be approximately one DBCO molecule per nanobody by monitoring the rection via the absorbance of DBCO at about 310 nm (Fig. S4f–h). The resulting αPD-L1-DBCO showed a similar binding affinity to PD-L1 to that of αPD-L1 (Fig. S4i and j). After incubation of the fMGL-labeled γδ T cells with αPD-L1-DBCO, flow cytometry and confocal fluorescence microscopy analysis revealed that αPD-L1 was conjugated on the surface of γδ T cells with high efficiency, yielding the αPD-L1-γδ T cells (Fig. 3b and c). The abundance of αPD-L1 covalently attached to γδ T cells was monitored with a previously reported method [16] (Fig. S4k). Approximately 6 × 10^7^ αPD-L1 molecules were labeled on one γδ T cell (Fig. S4l). By using flow cytometry to monitor the retention of the nanobody on the cell surface, we showed that even after 96 h, a significant amount of αPD-L1 remained on the γδ T cell surface (Fig. S5a). Collectively, all these data indicate that AMS-ManNAz-P-enabled cell surface engineering is a facile strategy to install functional antibody or nanobody on γδ T cells for the preparation of antibody–γδ T cell conjugates.

With the αPD-L1-γδ T cells in hand, we sought to evaluate whether they would bind to PD-L1-positive cancer cells and initiate the release of cytotoxic cytokines and cell killing (Fig. 3d). When co-culturing with OVCAR-8 cells, an PD-L1-overexpressed ovarian cancer cell line, αPD-L1-γδ T cells, but not WT γδ T cells, interacted with OVCAR-8 cells (Fig. 3e). Moreover, the αPD-L1-γδ T cells exhibited markedly higher cytotoxicity against OVCAR-8 cells than γδ T cells at varied effector-to-target ratios (Fig. 3f). The PD-L1 targeting-enhanced cytotoxicity of αPD-L1-γδ T cells was further confirmed in two other PD-L1-positive cancer cell lines, A2780-DDP and U251 cells (Fig. S5b and c). The killing of PD-L1-positive cancer cells by αPD-L1-γδ T cells was time-dependent and rapid, with marked cancer cell killing observed after 30 min of co-culture (Fig. 3g). On the other hand, the αPD-L1-γδ T cells remained active for killing cancer cells after culturing for 96 h (Fig. S5d). As indicated by the release of granzyme B (GrB) and interferon gamma (IFNγ), co-culture of αPD-L1-γδ T cells with OVCAR-8 cancer cells induced T cell activation (Fig. 3h and i). The activation of αPD-L1-γδ T cells was confirmed by the expression of cell-surface marker CD69 (Fig. S5e and f). For preparing αPD-L1-γδ T cells, fMGL with AMS-ManNAz-P resulted in αPD-L1-γδ T cells with higher cytotoxicity than 1,6-Pr_2_ManNAz (Fig. S5g).

To further validate that the enhanced cytotoxicity was via cell-surface engineering of γδ T cells with αPD-L1, cancer cell-killing efficiency of αPD-L1-γδ T cells was compared with SiaNAz-incorporated γδ T (N_3_-γδ T) cells, a mixture of γδ T cells with αPD-L1 and αCD7-γδ T cells (αCD7 as a negative control antibody). Except for αPD-L1-γδ T cells, all the others exhibited no more enhanced cytotoxicity than unengineered γδ T cells (Fig. 3j). Furthermore, blocking PD-L1 on the surface of OVCAR-8 cells with excess αPD-L1 abolished the enhanced cytotoxicity of αPD-L1-γδ T cells. In addition, overexpression of PD-L1 in MC38 cells enhanced the cell killing efficiency by αPD-L1-γδ T cells (Fig. S5h). By using HEK293T cells as a PD-L1-negative control, αPD-L1-γδ T cells exhibited much higher killing efficiency toward OVCAR-8 cells (Fig. S5i).

We then evaluated the αPD-L1-γδ T cells for killing primary cancer cells derived from patients with ovarian cancer. The cancer cells were isolated either from the primary lesions or ascites of the patients (Table  S1). The cultured primary cancer cells exhibited varied expression levels of PD-L1 (Fig. S6a–d). Upon co-culturing with αPD-L1-γδ T cells or γδ T cells, all the primary cells were lysed more efficiently by αPD-L1-γδ T cells (Fig. S6e–j). In addition, high levels of released GrB and IFNγ were observed in all primary ovarian cancer cells co-cultured with αPD-L1-γδ T cells (Fig. S6k–p). Notably, αPD-L1-γδ T cells could exert profound cytotoxicity toward the primary ovarian cancer cells, which expressed PD-L1 at relatively low levels among the six patients. We speculated that the PD-L1 on these cells are sufficient for binding to αPD-L1, thus enabling effective targeting by αPD-L1-γδ T cells. Taken together, these results demonstrate that cell-surface engineering of γδ T cells with targeting antibodies provides a versatile tool for enhancing the cytotoxicity and targeting specificity to cancer cell lines, as well as patient-derived primary cancer cells.

### Mechanism of cancer cell killing by αPD-L1-γδ T cells

**Figure 4.**
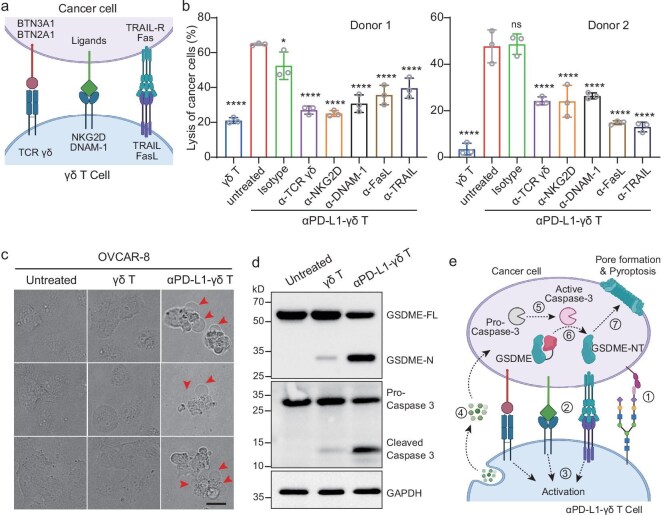
Pyroptosis of cancer cells induced by αPD-L1-γδ T cells. (a) Schematic representation of the known activation, co-stimulation and death receptors and ligands of γδ T cells. (b) Percentages of cancer cell-killing by αPD-L1-γδ T cells prepared from two different donors in the presence of neutralizing antibodies against indicated receptors or ligands. Data are presented as mean ± SD (*n* = 3). ns, not significant, *P < 0.05, ****P < 0.0001 (one-way ANOVA). (c) Bright-field microscopy images of OVCAR-8 cells treated with vehicle, γδ T cells or αPD-L1-γδ T cells. Three representative images are shown for each group. The red arrows indicate the bubble-like structures of pyroptotic cancer cells. Scale bar is 20 μm. (d) Western blot analysis on GSDME and caspase-3 in OVCAR-8 cells treated with vehicle, γδ T cells or αPD-L1-γδ T cells. GSDME-FL, full length GSDME; GSDME-N, the N-terminal domain of GSDME. The anti-GAPDH blot demonstrates comparable loading. (e) Proposed mechanism of αPD-L1-γδ T cells inducing pyroptosis of cancer cells.

Although the activation and cytotoxicity mechanisms of γδ T cells remain incompletely understood, activation induced by recognition of BTN3A1/BTN2A1 antigen by γδ TCRs, co-stimulation and death ligand signals have been proposed [1] (Fig. 4a). We then evaluated whether these activation, co-stimulation and death ligand signals play important roles in αPD-L1-γδ T-mediated cancer cell killing. Neutralizing antibodies targeting γδ TCRs, co-stimulation receptors NKG2D and DNAM-1, and death ligands FasL and TRAIL all efficiently impaired the killing of primary ovarian cancer cells by αPD-L1-γδ T cells (Fig. 4b). These results indicate that αPD-L1-γδ T cell cytotoxicity is dependent on the activation, co-stimulation and death ligand signals of γδ T cells.

When co-cultured with αPD-L1-γδ T cells, the bubble-like morphology was observed for OVCAR-8 cells (Fig. 4c). Moreover, the co-culture of OVCAR-8 and of αPD-L1-γδ T cells exhibited elevated release of ATP and HMGB1 (Fig. S7a and b). In addition, cleavage and release of the N-terminal domain of gasdermin E (GSDME) and cleavage and activation of caspase-3 were observed in the co-culture of OVCAR-8 and of αPD-L1-γδ T cells (Fig. 4d). Similar results were observed for two additional PD-L1-positive cancer cell lines, SK-OV-3 and U251 cells (Fig. S7c–g), and importantly in the patient-derived primary ovarian cancer cells (Fig. S7h and i). Taken together, these data demonstrate that the cancer cells, upon co-culturing with αPD-L1-γδ T cells, undergo pyroptosis [38,39].

We then asked whether the pyroptosis induced by αPD-L1-γδ T cells was dependent on the targeting antibody and antigen. By using a nanobody recognizing EGFR (αEGFR), we prepared the αEGFR-γδ T cells, which exhibited enhanced cytotoxicity toward A431 cells, an EGFR-positive human skin cancer cell line (Fig. S7j). The bubble-like morphology and cleaved GSDME were also observed, indicating pyroptosis of A431 cells (Fig. S7k and l). These results suggest that the chemically engineered γδ T cells induce cancer cell pyroptosis independent of conjugated antibodies and targeted tumor antigens.

On the basis of these results, we proposed that the targeting antibodies (e.g. αPD-L1) on the cell surface first facilitates binding of γδ T cells to the targeted cancer cells via binding to the tumor antigens (e.g. PD-L1), which induces the interactions between γδ TCR and BTN3A1/2A1, co-stimulation receptors and ligands, and death ligands and receptors. The γδ T cells are activated and release cytotoxic cytokines. In cancer cells, caspase-3 is in turn activated and cleaves GSDME to trigger pyroptosis (Fig. 4e).

### αPD-L1-γδ T cells exhibit enhanced anti-tumor activity *in vivo*

**Figure 5.**
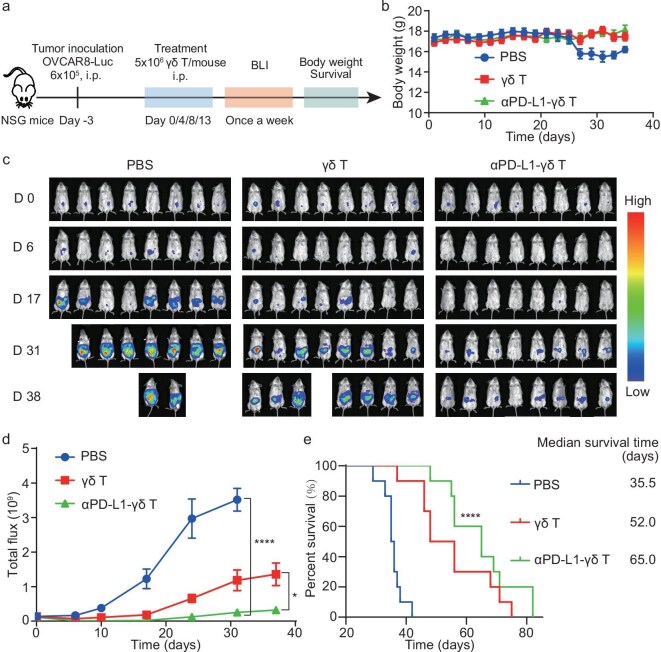
*In vivo* anti-tumor activity of αPD-L1-γδ T cells. (a) Schematic of the animal experiments. The immune-compromised NSG mice were xenografted with OVCAR8-Luc cells to establish the peritoneal metastasis model. The tumor-bearing mice were treated with vehicle, γδ T cells or αPD-L1-γδ T cells intraperitoneally every 4 days for a total of four times. The tumor burden was monitored by bioluminescence imaging (BLI) and the body weights and survivals were recorded. (b) Body weight curves of the tumor-bearing mice treated with vehicle, γδ T cells or αPD-L1-γδ T cells. (c) Representative time-course bioluminescence images of the tumor-bearing mice treated with vehicle, γδ T cells or αPD-L1-γδ T cells. (d) Quantitative analysis of the bioluminescence images in (c). Data are presented as mean ± SD (*n* = 10 mice per group). *P < 0.05, ****P < 0.0001 (one-way ANOVA). (e) Survival curves and the median survival times of the three groups of mice. *n* = 10 mice per group. **** *P* < 0.0001 [log-rank (Mantel-–Cox) test], αPD-L1-γδ T vs. PBS.

Ovarian cancer is the sixth most common gynecological tumor, with the highest mortality rate [40]. Bioinformatic analysis on ovarian cancer using a dataset deposited in Gene Expression Omnibus (GEO) [41] showed that higher infiltration of γδ T cells in ovarian cancer correlates with better prognosis (Fig. S7m). Clinically, ovarian cancer often develops peritoneal metastasis in the late stage, which greatly affects the prognosis of ovarian cancer patients [42]. We therefore sought to evaluate *in vivo* anti-tumor activity of αPD-L1-γδ T cells in the xenografted mouse model of peritoneal metastasis of ovarian cancer using OVCAR-8 cells stably expressing firefly luciferase (OVCAR8-Luc cells) (Fig. 5a). Since cell-surface sialic acids may be cleaved by extracellular sialidases *in vivo* [43], we first tested whether the cell-surface αPD-L1 conjugated with sialic acids were susceptible to sialidases. By using a recombinant sialidase that could efficiently remove cell-surface sialic acids with different linkages (Fig. S8a), we showed that αPD-L1 on αPD-L1-γδ T cells, as well as αHER2 on αHER2-γδ T cells, was resistant to sialidase cleavage (Fig. S8b and c). This was probably owing to the steric hindrance imposed by the antibodies.

The mice with OVCAR-8-Luc xenografts were treated intraperitoneally with vehicle, γδ T cells, or αPD-L1-γδ T cells every 4 days for a total of four doses. Tumor development and growth rate were monitored by bioluminescence imaging, with the body weight and survival rate recorded (Fig. 5a). Compared to the vehicle-treated mice, the mice treated with either γδ T cells or αPD-L1-γδ T cells better maintained their body weights (Fig. 5b). Bioluminescence imaging showed that αPD-L1-γδ T cells efficiently inhibited the growth of OVCAR-8 tumors, while γδ T cells exhibited a moderate inhibition (Fig. 5c and d). In agreement, unengineered γδ T cells were shown to exert *in vivo* anti-tumor activity [9]. The median survival time of the tumor-bearing mice were extended from 35.5 days to 52 days and 65 days when treated with γδ T cells and αPD-L1-γδ T cells, respectively (Fig. 5e). Furthermore, co-administration of γδ T cells with αPD-L1 did not improve the anti-tumor efficacy (Fig. S9). In addition, the αPD-L1-γδ T cells could also be intravenously administered, with anti-tumor efficacy similar to the intraperitoneally administered αPD-L1-γδ T cells (Fig. S10). Taken together, the data demonstrate that the αPD-L1-γδ T cells possess improved *in vivo* anti-tumor activity for PD-L1-positive tumors.

### Remodeling of the TME by αPD-L1-γδ T cells

**Figure 6.**
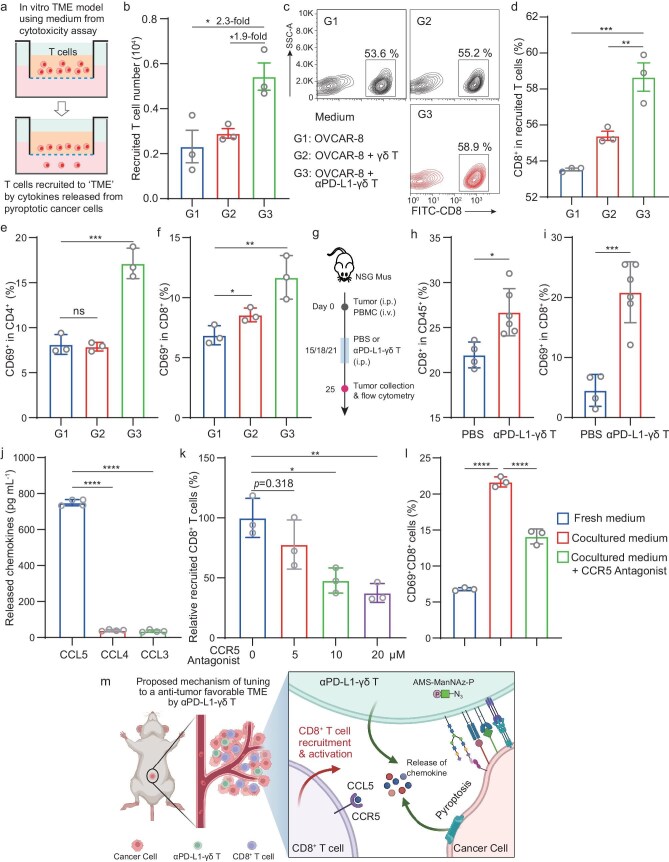
TME remodeling by αPD-L1-γδ T cells. (a) Transwell assay on the recruitment of T cells by TME mimics. (b) Number of recruited T cells by the medium from the OVCAR-8 cell culture, the αPD-L1-γδ T cell culture or the co-culture of αPD-L1-γδ T cells and OVCAR-8 cells. (c) Representative flow cytometry analysis of the recruited CD8^+^ T cells in (b). (d) Quantitative analysis of the flow cytometry data in (c). (e, f) Quantitative analysis of the activation of CD4^+^ T cells (e) and CD8^+^ T cells (f) incubated with indicated medium by flow cytometry. (g) Schematic of the mouse experiments to evaluate the TME remodeling by αPD-L1-γδ T cells. (h) Percentage of tumor-infiltrating CD8^+^ T cells among CD45^+^ cells. (i) Percentage of tumor-infiltrating CD8^+^ T cells with the activation phenotype. (j) Release of CCL3, CCL4 and CCL5 from the co-culture of αPD-L1-γδ T cells and OVCAR-8 cells. (k) CD8^+^ T cell recruitment in the presence of the CCR5 antagonist at varied concentrations. The CD8^+^ T cells were pretreated with the CCR5 antagonist for 30 min before the recruitment assay. (l) Activation of CD8^+^ T cells incubated with fresh medium, co-culture medium or coculture medium without the CCR5 antagonist. (m) Proposed mechanism of remodeling of the TME toward immune-active TME by αPD-L1-γδ T cells. Created with BioRender.com. In (b), (d), (e), (f) and (h–l), the data are presented as mean ± SD [*n* = 3 for (b), (d), (e), (f), (k) and (l); *n* = 4 for (j) and (h)-PBS group, and *n* = 6 for (h)-αPD-L1-γδ T group]. ns: not significant, *P < 0.05, **P < 0.01, ***P < 0.001, ****P < 0.0001 [unpaired Student's *t*-test in (h) and (i) and one-way ANOVA in the rest].

Considering the pyroptosis of cancer cells induced by antibody–γδ T cell conjugates (Fig. 4 and Fig. S7), we wondered whether it might lead to TME remodeling, as the roles of cancer cell pyroptosis in anti-tumor immunity have been demonstrated [44,45]. We set up a Transwell-based *in vitro* TME recruitment assay, in which the recruitment of T cells on the upper insert by the ‘TME mimic’ in the lower chamber was quantified (Fig. 6a). The culture medium from the co-culture of αPD-L1-γδ T cells and OVCAR-8 cells led to significant recruitment of T cells, showing 2.3- and 1.9-fold higher than the medium from OVCAR-8 alone and from the γδ T and OVCAR-8 co-culture, respectively (Fig. 6b). The medium from αPD-L1-γδ T and OVCAR-8 co-culture led to the recruitment of CD8^+^ T cells at a slightly higher proportion (Fig. 6c and d). Flow cytometry analysis showed that both CD4^+^ and CD8^+^ T cells were activated by the *in vitro* ‘TME mimic’ produced from the co-culture of αPD-L1-γδ T cells and OVCAR-8 cells (Fig. 6e and f).

The αPD-L1-γδ T cells were then administered into the mice xenografted with OVCAR-8 cells (Fig. 6g). Flow cytometry analysis on the tumor-infiltrating lymphocytes (TILs) revealed that upon αPD-L1-γδ T treatment, the infiltrated Vδ2^+^ γδ T cells were significantly increased (Fig. S11a–c). The tumorous γδ T cells were significantly activated (Fig. S11d) and mostly maintained a central memory phenotype with reduced proportion of exhaustion (Fig. S11e and f). The infiltration of CD8^+^ T cells was also increased upon αPD-L1-γδ T treatment (Fig. 6h and Fig. S11g), in agreement with the *in vitro* T cell recruitment results (Fig. 6a–d). The infiltrated CD8^+^ T cells were significantly activated (Fig. 6i) and also maintained central memory phenotype with a very small amount of exhaustion (Fig. S11h and i). Although infiltration of CD4^+^ T cells was slightly decreased (Fig. S11j and k), their activation remained at a high level with no significant alteration in the proportion of cells with the central memory and exhaustion phenotypes (Fig. S11l–n). Together, these results suggest a more tumor-suppressing TME induced by infiltration of αPD-L1-γδ T cells.

The C-C chemokine receptor type 5 (CCR5, also known as RANTES) plays an important role in TME remodeling by binding to chemokine ligands, such as C-C motif chemokine ligand 3/4/5 (CCL3/4/5) [46–48]. Co-culturing OVCAR-8 cells with αPD-L1-γδ T cells led to the release of a significant amount of CCL5, while the release of CCL3 and CCL4 was much lower (Fig. 6j). Blocking the CCR5–CCL5 interaction by a CCR5 antagonist maraviroc [49] significantly reduced CD8^+^ T cell recruitment (Fig. 6k). Moreover, the activation of CD8^+^ T cells by the co-culture medium of αPD-L1-γδ T cells and OVCAR-8 cells was hampered by inhibition of the CCR5–CCL5 interaction (Fig. 6l and Fig. S12a). In line with this, previous reports showed that CCL5 could activate T cells [50]. The CD8^+^ T cells were activated by CCL5 in a dose-dependent manner, which was suppressed by the CCR5 antagonist (Fig. S12b). These results, together, demonstrate that αPD-L1-γδ T cells not only possess improved cytotoxicity toward PD-L1-positive cancer cells, but also fine-tuned the TME, at least partially via the CCR5/CCL5 axis (Fig. 6m).

## CONCLUSION

In summary, we have developed a chemical strategy for engineering the cell surface of γδ T cells with targeting antibodies. Cell-surface sialic acids serve as an optimal anchoring site and can be metabolically labeled with azido sugars. The azido sialic acids are then conjugated with antibodies via click chemistry. As an example, αPD-L1-γδ T cells possess enhanced tumor-targeting efficiency and improved cytotoxicity towards PD-L1-positive cancer cells *in vitro* and tumors in living mice. It is worth noting that γδ T cells are non-immunogenic and can be allogenically transferred repeatedly. In principle, the efficacy of αPD-L1-γδ T cells can be further enhanced with more doses. Our work thus provides a platform for chemical engineering of γδ T cells with various functionalities, which holds great promise in promoting the development of adoptive cell therapy based on γδ T cells.

In regard to the clinical translation of the antibody–γδ T cell conjugates, several issues need to be further evaluated. For example, the quality control of the chemical engineering process (i.e. controlling the modification densities between different batches) should be optimized to ensure γδ T cell function and activity. Furthermore, different tumor antigens, in particular those with targeting antibodies in clinical use, can be exploited for targeting specific tumor types. In addition, the antibody–γδ T cell conjugates may be further equipped with on and off switches for further improving the specificity and efficacy.

## MATERIALS AND METHODS

### Expansion of γδ T cells

γδ T cells were expanded from healthy donor PBMCs (under the guideline of approved protocol: ICB00006761-M2023641). Briefly, PBMCs were isolated from whole blood by density-gradient centrifugation using Lymphocyte Separation Medium (Solarbio, P8610). PBMCs were resuspended and cultured in CTS OpTmizer T-Cell Expansion SFM medium (Gibco, A1048501) containing 10% fetal bovine serum (Gibco, 26010074) at a density of (1–3) × 10^6^ cells/mL. PBMCs were stimulated with 5 μM zoledronic acid (MCE, HY-13777) for the first 3 days and stimulated for 14 days with recombinant human interleukin-2 (T&L Biotechnology Co., Ltd., GMP-TL906-0050) at a concentration of 1000 IU/mL. Medium was supplemented every 2 days to maintain the cell density below 2 × 10^6^ cells/mL during the expansion. γδ T cell purity was validated at Day 14. Expanded cells were stained with anti-human CD3 (BioLegend, 317305) and anti-human TCR γ/δ (BioLegend, 331209) and analyzed by flow cytometry. γδ T cells with purity higher than 95% passed the quality control and were stored in liquid nitrogen for use.

### Metabolic glycoengineering and labeling of cells with DNA/protein/antibody

γδ T cells were seeded in a 48-well plate at a density of 5 × 10^4^ cells per well and incubated with indicated unnatural monosaccharides for indicated times. For the labeling of DNA/protein/antibody, γδ T cells after metabolic glycoengineering were reacted with DBCO-conjugated DNA (Azenta Life Sciences), protein (GFP) or antibody at a concentration of 10 μM in PBS for 30 min at 37°C. The efficacy of the labeling was determined by confocal microscopy imaging or flow cytometry.

## Supplementary Material

nwaf256_Supplemental_File
