# Supplementary material for: Chemical engineering of γδ T cells with cancer cell-targeting antibodies for enhanced tumor immunotherapy
Source: Natl Sci Rev. 2025 Jun 27;12(8):nwaf256. doi: 10.1093/nsr/nwaf256 (PMC12365757; doi:10.1093/nsr/nwaf256)
Supplement: nwaf256_Supplemental_File [file nwaf256_supplemental_file.pdf]

SUPPLEMENTARY DATA

Chemical engineering of γδ T cells with cancer cell-targeting antibodies for enhanced tumor immunotherapy

Long Chen[1,#*], Bo Cheng[2,3,4,#], Zhanqun Yang[1,#], Mengzhu Zheng[5,#], Tianyu Chu[2,3], Pan Wang[6], Tianhui He[6], Yuan Xue[1], Houyi Ren[1], Liting Zheng[7], Peng Zhou[8], Xiaxuan Li[5], Haichuan Zhu[9], Hongyan Guo[6*], Xing Chen[2,3,10,11,12*], Jian Lin[1,5,11,13*]

[1]Department of Pharmacy, Peking University Third Hospital Cancer Center, Peking University Third Hospital, Beijing 100191, China

[2]College of Chemistry and Molecular Engineering, Peking University, Beijing 100871, China

[3]Beijing National Laboratory for Molecular Sciences, Peking University, Beijing 100871, China

[4]School of Pharmaceutical Sciences, Peking University, Beijing 100191, China

[5]Key Laboratory of Tropical Biological Resources of Ministry of Education, Song Li's Academician Workstation of Hainan University, School of Pharmaceutical Sciences, Hainan University, Haikou 572000, China

[6]Department of Obstetrics and Gynecology, Peking University Third Hospital, Beijing 100191, China

[7]Artificial Auditory Laboratory of Jiangsu Province, Xuzhou Medical University, Xuzhou 221004, China.

[8]LinXCell Biotechnologies, Beijing 102600, China

[9]School of Life Science and Health, Wuhan University of Science and Technology, Wuhan 430081, China

[10]Peking-Tsinghua Center for Life Sciences, Peking University, Beijing 100871, China

[11]Synthetic and Functional Biomolecules Center, Peking University, Beijing 100871, China

[12]Key Laboratory of Bioorganic Chemistry and Molecular Engineering of Ministry of Education,

[13]College of Life Science, Anhui Medical University, Hefei 230032, China

[*]e-mail: lcchenlong@163.com; bysyghy@163.com; xingchen@pku.edu.cn; linjian@pku.edu.cn

[#]These authors contributed equally: Long Chen, Bo Cheng, Zhanqun Yang, Mengzhu Zheng

SUPPLEMENTARY MATERIALS AND METHODS

Expression, purification and labeling of Anti-PD-L1 nanobody

Anti-PD-L1 (αPD-L1) nanobody was expressed and purified from *E. coli* according to literature[1]. Briefly, Anti-PD-L1 was first expressed in *E. coli* BL21 strain with C-terminal His-tag. The bacteria were lysed by sonication and then purified with HisTrap column (Cytiva, 17524802). Purified protein was buffer-changed to PBS and stored at -80 °C before use. Anti-PD-L1 nanobody (50 μM) reacted with DBCO- NHS (MCE, HY-140272, 250 μM) at 30 °C for 30 min and then was purified by flow through a PD-10 column (Cytiva, 17085101). DBCO-labeled Anti-PD-L1 was stored at -80 °C before use for cell labeling. For the labeling of anti-HER2 antibody (Trastuzumab, Henlius) similar conditions were adopted.

Intact glycopeptides mass spectrometry analysis

γδ T cells were metabolically labeled with 100 μM AMS-ManNAz-P for 48 hours before harvested. Cells were collected and washed with PBS (pH 7.4, Gibco) for three times. After the last wash, cells were resuspended and lysed in 4% sodium dodecyl sulfate in H_2O (4% SDS, wt/vol). After being ultrasonically crushed on ice for 1 min and heated at 95 °C for 10 min, the samples were centrifuged at 20,000 *g* for 20 min and the supernatants were collected. Protein concentrations of the cell lysis were determined by Rapid Gold BCA Protein Assay Kit (Pierce) and diluted to 5 mg/mL uniformly using 4% SDS.

8 mg proteins from γδ T cell lysis were incubated with 100 μM (final concentrations) alkyne-PC-biotin (Confluore, Cat. No. BCP-19), 400 μM $CuSO_4$, 800 μM BTTAA (Confluore, Cat. No. BDJ-4), and 2.5 mM freshly prepared sodium ascorbate and the volume of the solution was supplemented up to 6 mL volume by PBS. The click chemistry reaction system was mixed well and reacted at 25 °C for 3 h in the dark. The resulting mixture was precipitated by methanol ($V_{sample}/V_{methanol}$ 1:8) at -80 °C overnight.

After being washed three times with pre-cooled methanol, the proteins were resuspended in a solution containing 4 M urea (Sigma-Aldrich) and 50 mM ammonium bicarbonate (ABC, Sigma-Aldrich) at a concentration of 4 mg/mL. In order to reduce disulfide bonds, the proteins were treated with 10 mM dithiothreitol (DTT, Aladdin) at 37 °C for 45 min in the dark. Next, the proteins were incubated with 20 mM iodoacetamide (IAA, Psaitong) at 25 °C for 45 min in the dark to block free cysteines of proteins. Then the urea concentration of the system was diluted to 0.8 M using 50 mM ABC and the solution was subsequently incubated with mass spectra grade trypsin (Promega) at an enzyme-to-substrate ratio of 1:50 at 37 °C for 20 h in the dark.

The tryptic peptides were mixed with Streptavidin agarose beads (10 μL beads for 1 mg peptides,

Thermo Fisher Scientific, Cat No. 20353) at 25 °C with continuous rotation for 3 h in the dark. After being washed by PBS for 5 times and then pure H_2O for 5 times, the beads were resuspended in 200 μL 0.1% formic acid (Sigma-Aldrich) in H_2O (0.1% FA, vol/vol), followed by 365 nm UV cleavage for 15 min on ice twice using a UV crosslinker (CL-1000 UV Crosslinker; UVP). The supernatant was collected, evaporated in a vacuum centrifuge, and subjected to LC-MS/MS analysis.

LC-MS/MS analysis of enriched glycopeptides was performed on the Orbitrap Fusion Lumos Tribrid Mass Spectrometer (Thermo Fisher Scientific) equipped with a C18 capillary column (75 μm × 15 cm). The peptides were dissolved in 0.1% FA, separated with a gradient of 360 min by the UltiMate 3000 HPLC system (Thermo Fisher Scientific) and then subjected to the electron-transfer/higher-energy collisional dissociation-based MS/MS system with the sceHCD-pd-EThcD method as reported by Click-iG technique[2]. Under the positive ion mode, full-scan mass spectra were obtained over the range from 350 to 2,000 m/z, with the resolution of 120,000 at 200 m/z. Higher-energy collisional dissociation with stepped collision energy (sceHCD) was set as 30±10. The presence of specific sugar oxonium ions (m/z = 168.0654, 186.0760, 204.0865, 274.0921, 292.1027, 300.1302, 329.1455, 366.1395, 388.1463, 399.1992, 405.2130) in the sceHCD spectra was used to trigger EThcD fragmentation of corresponding precursor ions with the supplemental activation of 35 and a resolution setting of 30,000 at 200 m/z.

Raw data from LC-MS/MS identification were analyzed by pGlyco software (version 3.1) which was downloaded on GitHub (https://github.com/pFindStudio/pGlyco3/releases)[3]. The parameters were set as follows and the sections not mentioned were kept as default. The fragmentation mode was chosen as "HCD+ETHCD" according to the mass spectrometry method. The SwissPort Homo sapiens database was downloaded from Uniprot (https://www.uniprot.org) and set as the fasta file for protein sequence matching. "pGlyco-N-human.gdb" was chosen as the glycan database for N-glycan analysis and "Multi-Site-O-Glycan.gdb" for O-glycan analysis. Monosaccharide HexNAc labeled as "N" in pGlyco software, standing for GalNAc or GlcNAc, was variably modified as "PG", a name already built in pGlyco which stands for the residual structure of HexNAc labeled by azide, reacted with alkyne-PC-biotin and cleaved by UV. Similarly, "A" standing for Neu5Ac in pGlyco was variably modified as "S" which meant the residual structure of Neu5Ac labeled by azide, reacted with alkyne-PC-biotin and cleaved by UV. The maximum number of variable modifications allowed on glycan structures was set as 2 and the corresponding modified glycan database was allowed to expand up to 10,000,000. The representative MS spectra were annotated by gLable software, which was a supplementary section in the package of pGlyco3 software.

Confocal microscope and flow cytometry analysis of metabolic glycoengineering and cell

labeling with DNA/Protein/Antibody

To verify the levels of metabolic glycoengineering, cells were first incubated with different unnatural monosaccharides at indicated times and concentrations. Cells were washed and incubated with 100 μM of DBCO- Biotin (MCE, HY-130809) at room temperature for 15 minutes, followed by incubation with 2 μg/mL of Streptavidin Alexa Fluor™ 488 Conjugate (Invitrogen, S11223) at 4 °C for 15 minutes. Cells were then washed and stained with Hoechst 33342 for imaging or without Hoechst 33342 staining for flow cytometry.

To verify the efficacy of αPD-L1 labelling, αPD-L1-γδ T cells stained with 2 μg/mL of biotinylated Human recombinant PD-L1 protein (Sino Biological, 10084-H49H-B) at room temperature for 15 minutes, followed by incubation with 2 μg/mL of Streptavidin Alexa Fluor™ 488 Conjugate at 4 °C for 15 minutes. Cells were then washed and stained (or non-stained) with Hoechst 33342 for imaging or flow cytometry. To verify GFP labeling, GFP-γδ T was stained (or non-stained) with Hoechst 33342 for imaging or flow cytometry. To verify DNA aptamer labeling, Aptamer-γδ T was stained with 2 μg/mL of Streptavidin Alexa Fluor™ 488 Conjugate (Invitrogen, S11223) at 4 °C for 15 minutes and stained (or non-stained) with Hoechst 33342 for imaging or flow cytometry.

The images were taken using a Zeiss confocal microscope (Zeiss LSM900) in the Hoechst and GFP channels. Flow cytometry was conducted on Bio-Rad S3e (Bio-Rad, Brea, CA, USA) and data was analyzed by FlowJo software.

Validation of S-glyco modification

γδ T cells were resuspended in ice-cold PBS and sonicated in ice. Cell lysates were collected by centrifugation (20,000 *g*, 10 min) at 4 °C. Cell lysate protein concentration was determined by using the Pierce™ BCA Protein Assay Kit (ThermoFisher, 23227). 50 μL cell lysates (1 mg/mL) were incubated with 2 mM ManNAz, AMS-ManNAz-P or Ac_4ManNAz at 37 °C for 2 h. The resulting lysates were precipitated by 400 μL pre-cooled methanol at -80 °C for 1 h. The precipitated proteins were centrifuged at 8,000 *g* for 5 min at 4 °C and washed twice with 500 μL cold methanol. The precipitated proteins were resuspended at 50 μL PBS containing 0.4% SDS, 100 μM DBCO-Cy3 (Lumiprobe, A10F0) for 1h at 37 °C. The reaction mixtures were subjected to SDS-PAGE and S-glyco modification was visualized by in-gel fluorescence using ChemiDoc System (Bio-Rad).

KEGG and GO enrichment of the S-glyco modification labeled proteins

S-glyco modification labeled proteins were acquired from the previous report[4] and subjected to KEGG and GO enrichment analysis. We performed enrichment analysis using the enrichKEGG and enricher functions from the R package clusterProfiler. The KEGG terms were obtained from the

KEGG database (https://www.kegg.jp/), while the GO terms were obtained from the C5 ontology gene sets in the MSigDB database (only retaining GO:BP-related terms) after filtering.

Validation of the binding of αPD-L1-γδ T to PD-L1 positive cancer cells

To confirm the enhanced binding of αPD-L1-γδ T to PD-L1 positive cancer cells, PD-L1 positive cancer cells were labelled with CSFE (Invitrogen, 65-0850-84) and seeded in 48-well plates. After an overnight culture, Hoechst 33342-labeled γδ T or αPD-L1-γδ T were added and incubated for 1 hour. Unbonded cells in the cell culture medium was discarded and the cells remained in the wells were washed 3 times with PBS. The images were taken using fluorescence microscope in the Hoechst and CFSE channels.

RhoA activity assay

20 μg RhoA protein (Beyotime, P2063) was incubated with 2 mM $Ac_4ManNAz$ or AMS-ManNAz-P for 2 hours at 37 °C in 20 μL volume, and enzyme activity was detected using a GTPase-Glo^{TM} Assay (Promega, V7682) according to manufacturer's instruction. Briefly, 5 μL RhoA reaction mixture was added with 5 μl of the 2 X GTP solution and incubated at room temperature for 90 min. 10 μl of reconstituted GTPase-Glo™ Reagent was then added, mixed briefly and incubate with shaking for another 30 min at room temperature. 20 μl of Detection Reagent was added and incubated for 5 min at room temperature. Luminescence was detected with a plate-reader.

T cell activation assay

γδ T cells were pre-treated with unnatural monosaccharides, ManNAz (1 mM), AMS-ManNAz-P (200 μM), $Ac_4ManNAz$ (200 μM) or 1,6-$Pr_2ManNAz$ (200 μM) for 24 h. Cells were then harvested and washed with 1% FBS and stained with PerCP/Cyanine5.5-conjugated anti-CD25 (BioLegend, 302626) for 30 min at 4 °C. Cells were then washed with 1% FBS and γδ T cell activation was analyzed by flow cytometry (Bio-Rad S3e).

Binding affinity measurement

Binding affinity of αPD-L1 and DBCO-αPD-L1 to PD-L1 was performed using ForteBio Octet red 96e. Briefly, recombinant biotinylated human PD-L1 protein (SinoBiological, 10084-H49H-B) was loaded onto streptavidin tips and then titrated with various concentrations of αPD-L1 or DBCO-αPD-L1. Dissociation constants were determined by fitting the association and dissociation curves.

In vitro cytotoxicity assay

In vitro cytotoxicity of γδ T and αPD-L1-γδ T cells to cancer cells was measured using the Non-radioactive Cytotoxicity Assay kit (Promega, G1780) according to manufacturer's instructions. Briefly, target cells (cancer cells) were first seeded in 96-well plates. Effector cells (γδ T and αPD-L1-γδ T) at various effector-to-target (E:T) ratios (0.5:1, 1:1, and 2:1) was then added and incubated for 3 hours at 37 °C. Cytotoxicity of αHer2-γδ T was measured similarly, with effector-to-target ratio set at 1:1 for MDA-MB-231 cells and 2:1 for MDA-MB-453 cells. Cytotoxicity was determined by LDH release and calculated as: Cytotoxicity % = LDH (experiment group release) / LDH (maximum release) * 100%.

Measurement of cytokine release

PD-L1 positive OVCAR-8 cells were cocultured with γδ T or αPD-L1-γδ T cells at an effector-to-target of 1:1 for 3 hours at 37 °C. Cell culture supernatants were collected and Granzyme B and IFNγ released to the medium was determined using ELISA (Solarbio, SEKH-0193, SEKH-0046).

Primary ovarian cancer cell

Primary ovarian cancer cells were isolated either from the ascitic fluid or the primary lesion of ovarian cancer patients with ethical approval by the Ethics Committee of Peking University Third Hospital (ID: IRBO0006761-M2019291). Primary cancer cells were maintained in RPMI 1640 medium supplemented with 10% fetal bovine serum (Gibco, 26010074) and 100 U/mL penicillin/streptomycin (Gibco, 15140122).

Antibody neutralization assay

Antibody neutralization assay was conducted with modification to the in vitro cytotoxicity assay. Briefly, αPD-L1-γδ T cells was pre-treated with or without 20 μg/mL of isotype control, or neutralizing anti-human TCR γ/δ (BioLegend, 331202), anti-human NKG2D (BioLegend, 320805), anti-human DNAM-1 (Abcam, ab33397), anti-human FasL (BioLegend, 306407), anti-human TRAIL (BioLegend, 308206) for 30 minutes to pre-block corresponding receptors or ligands. The pre-blocked αPD-L1-γδ T cells were subjected to in vitro cytotoxicity assay with effector-to-target ratio set at 1:1. Cytotoxicity was determined by LDH release.

Western blot analysis

Western blot was performed according to standard protocol. Anti-GSDME antibody (Abcam, ab215191), Anti-cleaved Caspase-3 antibody (Abcam, ab32042) and GAPDH (Earthox, 718022)

served as primary and were used at 1:1000 dilution. HRP-conjugated goat anti-rabbit secondary antibody (Earthox, E030120-01) at 1:5000 dilution was used for signal visualization. Images were taken using the BLT GelView system (Guangzhou Bo Lu Teng Biotechnology Co., Ltd., GelView 6000Plus).

Sialidase cleavage experiments

γδ T cells, αPD-L1-γδ T, and αHer2-γδ T cells were collected and washed twice with PBS. The cell density was adjusted to 1×10^6 cells/mL, and sialidase α-2-3, 2-6, 2-8-Sialidosidase (SpNanA)[5] was added to a final concentration of 10 μg/ml in PBS. The mixture was incubated at 37 °C for 30 min to cleave sialic acids on the cell surface. After incubation, the cells were centrifuged at 400 *g* for 5 min to terminate the reaction. Streptavidin-BF647 (Bioss, bs-0437P-BF647) labeled tetrameric glycan recombinant affinity binders (tetra-GRABs-BF647)[5], PE anti-His Tag Antibody (BioLegend, 362603) and PE anti-human IgG Fc Antibody (BioLegend, 410707) were used to detect cell surface sialic acids, anti-PD-L1 nanobody and anti-HER2 antibody before and after sialidase treatment, respectively, by flow cytometry.

Analysis of γδ T cell infiltration and patients' survival in ovarian cancer

The RNA-seq and clinical data of 270 ovarian cancer patients were obtained from GSE32062 (https://www.ncbi.nlm.nih.gov/geo/query/acc.cgi?acc=GSE32062) . CIBERSORT was utilized for the analysis of immune cell composition based on the gene expression data. CIBERSORT was executed with the following settings: LM22 signature gene file, 1000 permutations, and quantile normalization was allowed. Subsequently, we selected 46 samples with non-zero T cell gamma delta cell scores. Using the surv_cutpoint function from the survminer package, we determined the cutoff value and generated survival curves using the ggsurvplot function.

Animal experiments

All animal experiments were approved by and under the guideline of IACUC of Peking University Health Science Center, Beijing, China (No. A2023016). NSG mice (female, 6-8 weeks, Speiford Beijing Biotechnology Co., Ltd.) were inoculated intraperitoneally (i.p.) with OVCAR8-Luc cells (6×10^5) on day -3. At day 0, 4, 8 and 13, 5×10^6 γδ T or αPD-L1-γδ T was injected to the xenograft mice intraperitoneally or intravenously. For the experiment shown in Figure S10, αPD-L1 corresponding to the amount of αPD-L1 labeled on γδ T (calculated from Figure S4m) was administrated alone or co-administrated with γδ T cells intraperitoneally. Tumor burdens were verified every week by bioluminescence imaging (Guangzhou Bo Lu Teng Biotechnology Co., Ltd.,

AniView). Body weights were monitored every two days. And mice survival was also monitored.

Transwell-based in vitro T cell recruitment assay

Cell culture medium supernatant from in vitro cytotoxicity assay (E:T ratio 1:1) was collected and added to the lower chamber of Transwell (Corning, 3421). T cells (2×10^5/mL) were added to the upper insert. Cells were incubated at 37 °C for 6 h. Numbers of T cells recruited to the lower chamber was determined using CCK8 (MedChemExpress, HY-K0301). Recruited T cells were also analyzed by flow cytometry to determine the fraction of $CD8^+$ T (BioLegend, 344703) being recruited. For CCR5 related recruitment, $CD8^+$ T cells were pre-blocked with different concentrations of CCR5 antagonist (Maraviroc, MedChemExpress, HY-13004) for 30 minutes before start of the recruitment assay.

In vitro T cell activation by medium from cytotoxicity assay

Purified human primary T cells were incubated with cell culture medium supernatant from in vitro cytotoxicity assay (E:T ratio 1:1) for 24 hours. T cells were then stained with anti-CD8 (BioLegend, 344703) or anti-CD4 (BioLegend, 980802) and anti-CD69 (BioLegend, 985202). The activation of $CD8^+$ and $CD4^+$ T cells was determined with flow cytometry.

Flow cytometry to validate T cell infiltration

NSG mice (female, 6-8 weeks, Speiford Beijing Biotechnology Co., Ltd.) were inoculated intraperitoneally (i.p.) with OVCAR8-Luc cells (6×10^5) on day 0. At the same day 1×10^7 purified human PBMC was injected intravenously. At day 15, 18 and 21, 5×10^6 αPD-L1-γδ T was injected to the xenograft mice intraperitoneally. At day 24, mouse was sacrificed and tumor tissues were collected. The tumor tissues were dissociated using the tumor tissue dissociation kit (Miltenyi, 130-095-929), then passed through a 70 μm filter (Falcon, 352350) to obtain a single-cell suspension. After lysing red blood cells with the red blood cell lysis solution (Solarbio, R1010), the cells were washed with 1% FBS and centrifuged at 350 *g* for 5 minutes to collect cells. Subsequently, the cells were stained with the following antibodies: Zombie Aqua™ Fixable Viability Kit (BioLegend, 423101), Brilliant Violet 421™-conjugated anti-CD45 (BioLegend, 368521), FITC-conjugated anti-CD4 (BioLegend, 300505), APC/Fire™ 750-conjugated anti-CD8 (BioLegend, 344745), PE-conjugated anti-Vδ2 TCR (BD Pharmingen, 555739), Brilliant Violet 605™-conjugated anti-CD69 (BioLegend, 310937), APC-conjugated anti-PD1 (BioLegend, 621609), PE/Cyanine7-conjugated anti-CD44 (BioLegend, 338815), and Brilliant Violet 711™-conjugated anti-CD62L (BioLegend, 304895) for 30 minutes. After incubation, cells were washed with 1% FBS. T cell infiltration and

activation were then analyzed by flow cytometry (SONY ID7000).

Statistical analysis

All statistical analyses were performed using GraphPad Prism 9. The statistical significance was assessed by an unpaired one-tailed Student's *t*-test, one -way ANOVA test or Log-rank (Mantel-Cox) test. P values less than 0.05 were considered significant. Data with error bars represent mean ± SD or SEM.

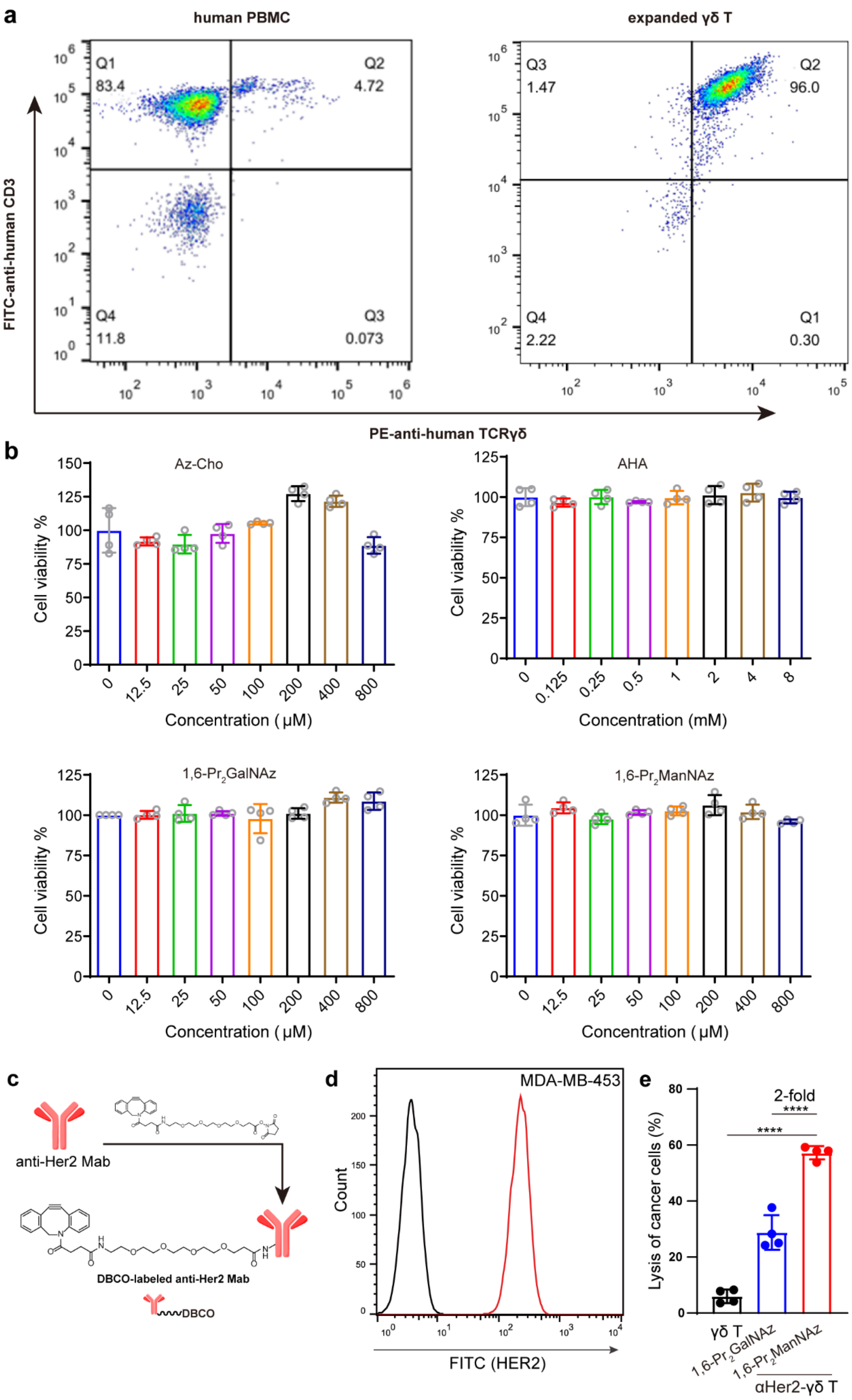
a
human PBMC
expanded γδ T
Q1
83.4
Q2
4.72
Q4
11.8
Q3
0.073
Q3
1.47
Q2
96.0
Q4
2.22
Q1
0.30
FITC-anti-human CD3
PE-anti-human TCRγδ
b
Az-Cho
AHA
1,6-Pr2GalNAz
1,6-Pr2ManNAz
Cell viability %
Concentration (μM)
Concentration (mM)
c
anti-Her2 Mab
DBCO-labeled anti-Her2 Mab
DBCO
d
MDA-MB-453
Count
FITC (HER2)
e
Lysis of cancer cells (%)
2-fold
****
****
γδ T
1,6-Pr2GalNAz
1,6-Pr2ManNAz
αHer2-γδ T

Figure S1. Preparation and characterization of αHer2-γδ T cells. (a) Flow cytometry analysis of human PBMCs and expanded γδ T cells, which were stained with antibodies against CD3 and TCRγδ. (b) Cell viability of γδ T cells incubated with Az-Cho, AHA, 1,6-Pr_2GalNAz, or 1,6-Pr_2ManNAz at varied concentrations, as shown by the CCK-8 assay. (c) Schematic showing the procedures for preparing αHer2-DBCO. (d) Representative flow cytometry histogram showing MDA-MB-453 cells stained with αHer2-FITC. (e) Percentages of killed MDA-MB-453 cells incubated with γδ T cells or αHer2-γδ T cells at the 1:2 ratio for 3 h. The αHer2-γδ T cells were prepared by labeling with 200 μM 1,6-Pr_2GalNAz or 200 μM 1,6-Pr_2ManNAz. Data are presented as mean ± SD (n=4). **** $p < 0.0001$ (one-way ANOVA).

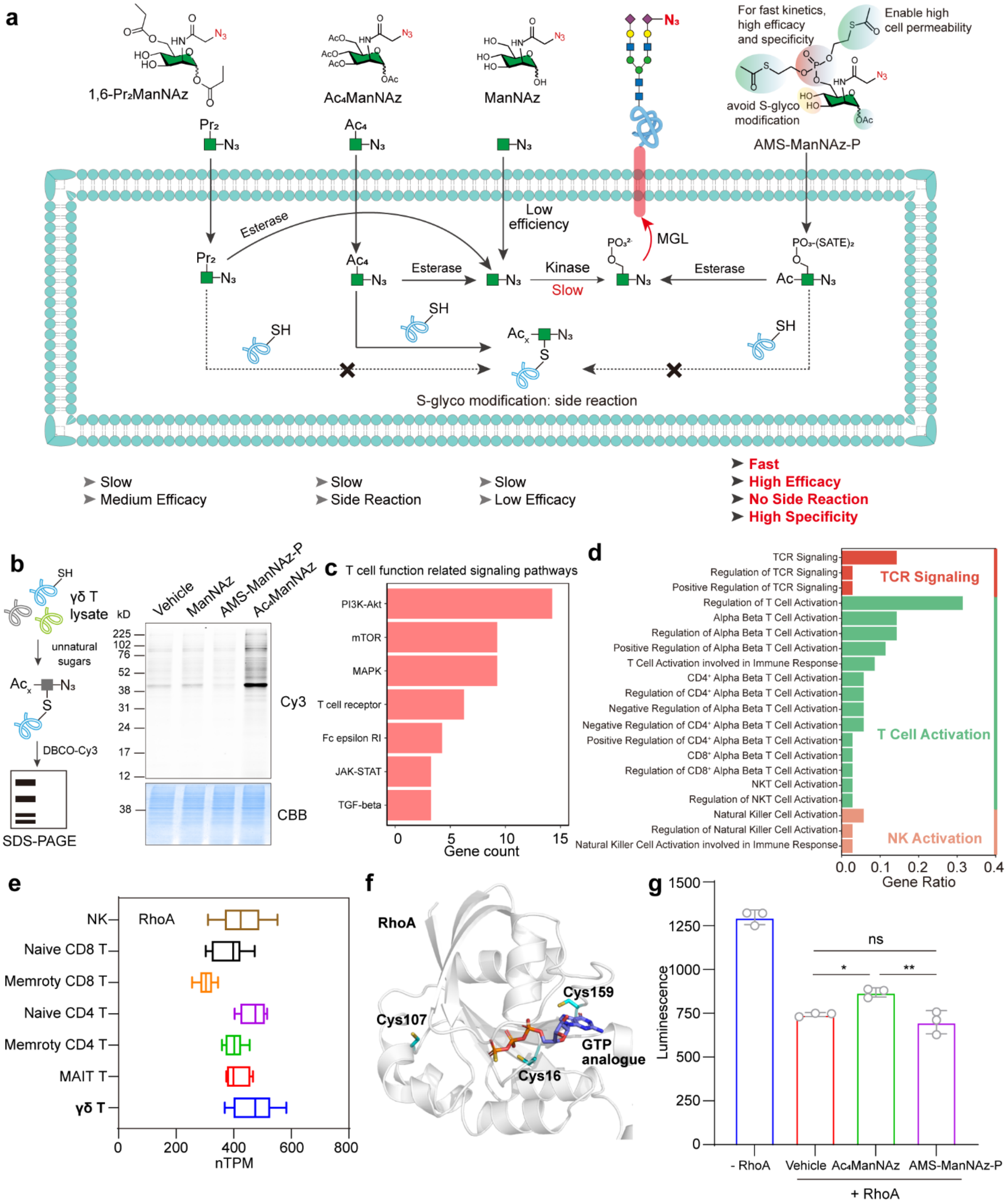

Figure S2. Evaluation of unnatural sugars in γδ T cells. (a) Unnatural sugars used for metabolically labeling cell-surface sialic acids, with their specificity and efficiency summarized. (b) Cell lysate assay for *S*-glyco-modification. The SDS-PAGE gel shows the lysates of γδ T cells directly incubated with vehicle, 2 mM ManNAz, 2 mM AMS-ManNAz-P, or 2 mM $Ac_4ManNAz$. (c) KEGG analysis of the T cell function-related pathways that were enriched with proteins modified by *S*-glyco-modification. (d) GO molecular function enriched with proteins modified by *S*-glyco-modification.

(e) mRNA expression levels of RhoA in NK and various T cells. nTPM, transcripts per million. The data were acquired from the Human Protein Atlas. (f) Structure of RhoA in complex with GTP analogue (PDB code 1A2B). Three cysteine residues modified by $Ac_4ManNAz$ are shown. (g) In vitro luminescence assay showing the enzymatic activity of RhoA in the presence of vehicle, $Ac_4ManNAz$, or AMS-ManNAz-P. The data are presented as mean ± SD (n=3). ns, not significant, $^*p < 0.05$, $^{**}p < 0.01$ (one-way ANOVA).

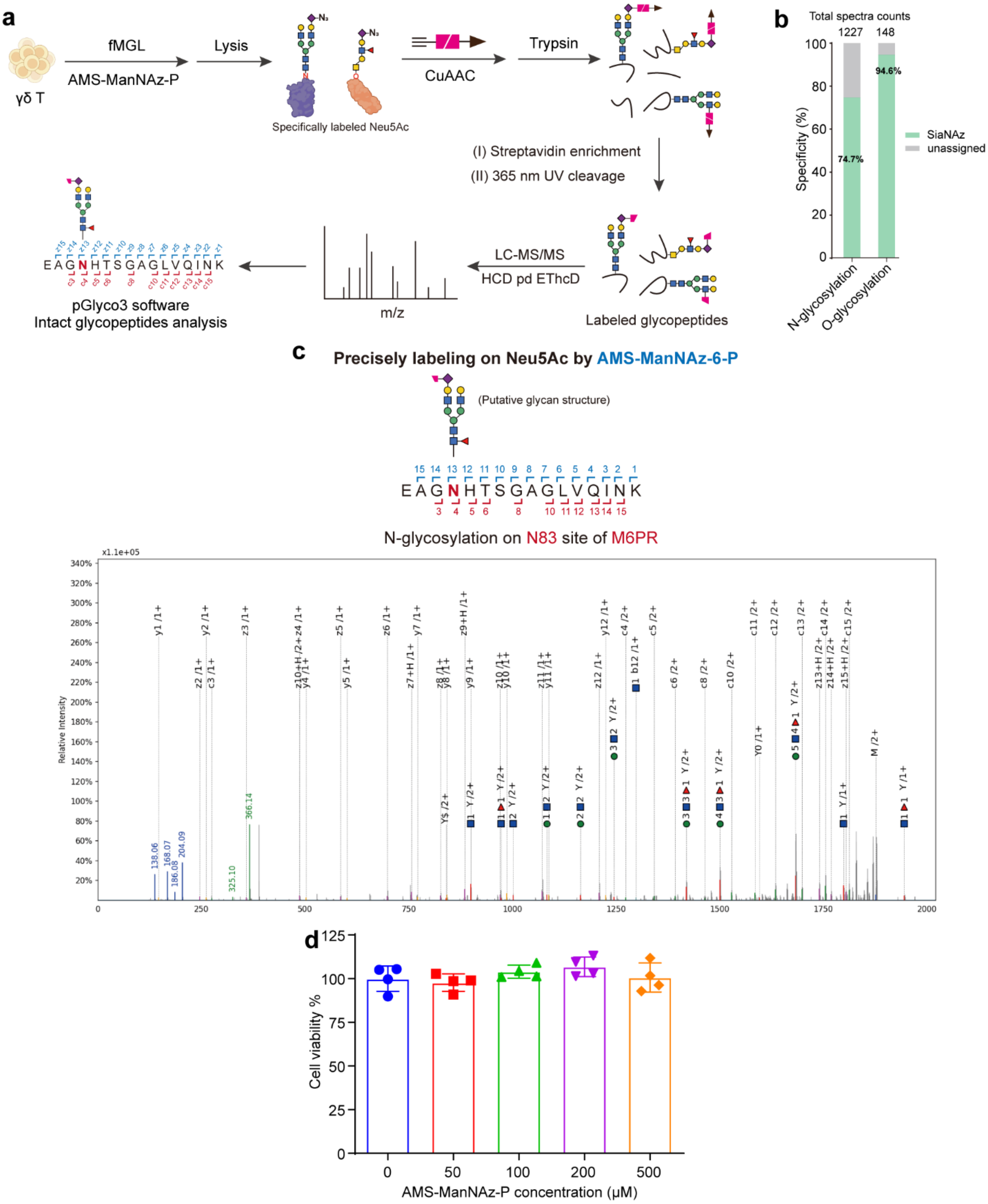

Figure S3. Specific labeling of sialic acids with no cytotoxicity in γδ T cells by AMS-ManNAz-P. (a) Procedures of profiling of intact glycopeptides in γδ T cells by click-iG using AMS-ManNAz-P. (b) Numbers of *N*-glycosylated and *O*-glycosylated peptide spectral matches (PSMs) containing SiaNAz. (c) Representative MS2 spectrum from the *N*-glycopeptide EAGNHTSGAGLVQINK with the *N*-glycan at N83 incorporated with SiaNAz. (d) Cell viability of γδ T cells treated with AMS-ManNAz-P at varied concentrations for 24 h, as assayed by the CCK-8 assay.

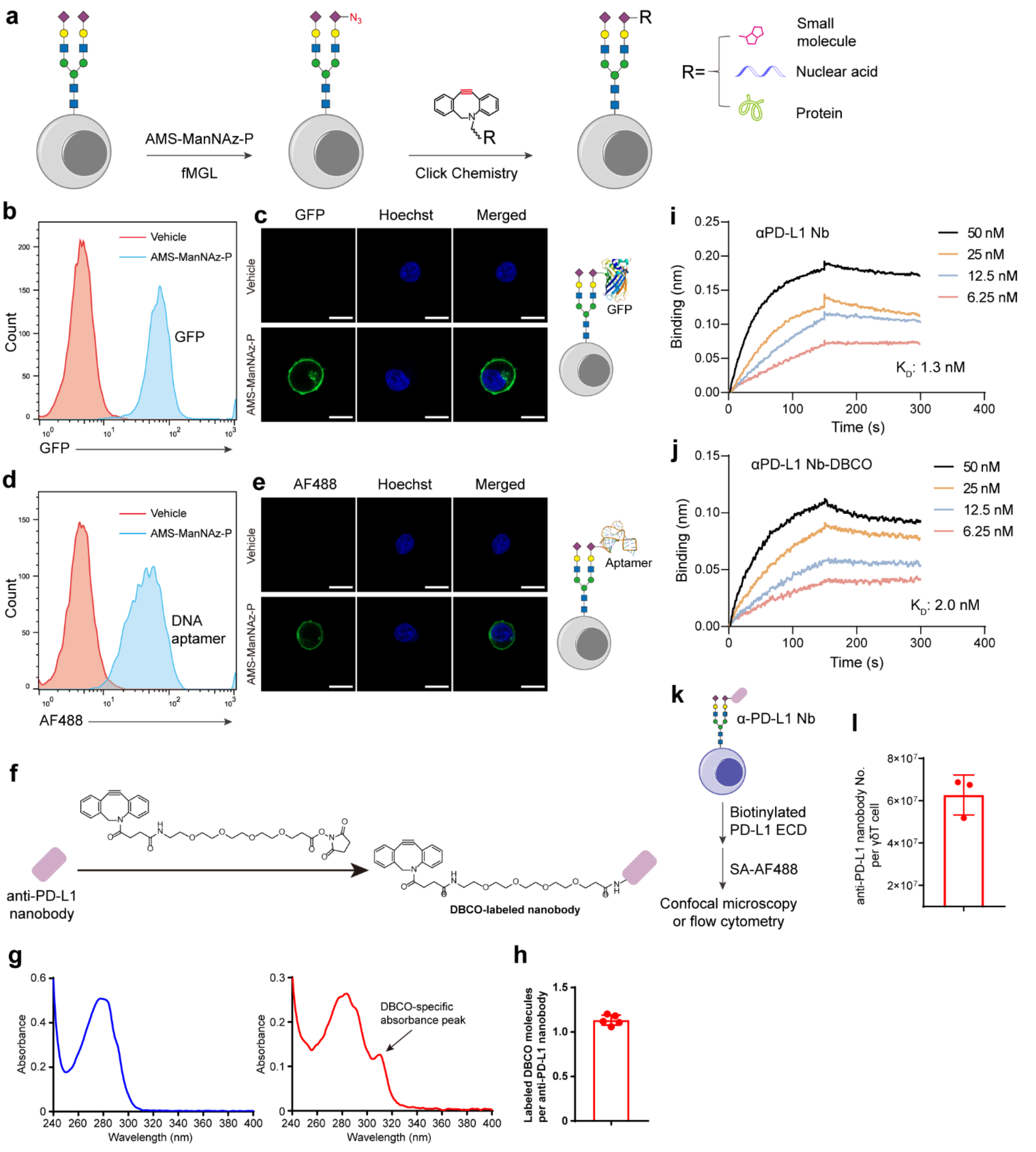

Figure S4. AMS-ManNAz-P-enabled cell-surface engineering. (a) γδ T cells are metabolically incorporated with SiaNAz via fMGL and subsequently conjugated with various functionalities via click chemistry. (b,c) Flow cytometry analysis (b) and confocal fluorescence microscopy images (c) of γδ T cells incubated with vehicle or 200 μM AMS-ManNAz-P for 24 h, followed by reaction with DBCO-GFP. (d,e) Flow cytometry analysis (d) and confocal fluorescence microscopy images (e) of γδ T cells incubated with vehicle or 200 μM AMS-ManNAz-P for 24 h, followed by reaction with

DBCO-aptamer conjugated with biotin. The cells were than stained with streptavidin-AF488. (f) αPD-L1 is functionalized with DBCO via lysine chemistry. (g) Representative UV-Vis absorbance spectra of αPD-L1 (left panel) and αPD-L1-DBCO (right panel). The inserted arrow indicates the DBCO-specific peak. (h) Quantitative analysis of the number of DBCO molecules per αPD-L1. (i,j) Biolayer Interferometry (BLI) curves of αPD-L1 (i) and αPD-L1-DBCO (j) binding to PD-L1. The determined K_D were 1.3 nM and 2.0 nM, respectively. (k) Schematic of the validation strategy of αPD-L1-γδ T cells. PD-L1 ECD: recombinant extracellular domain of PD-L1. (l) Quantitative analysis of the number of αPD-L1 molecules per γδ T cell. In c and e, scale bars, 20 μm. In h and l, data are presented as mean ± SD (n=3 for l and 5 for h)

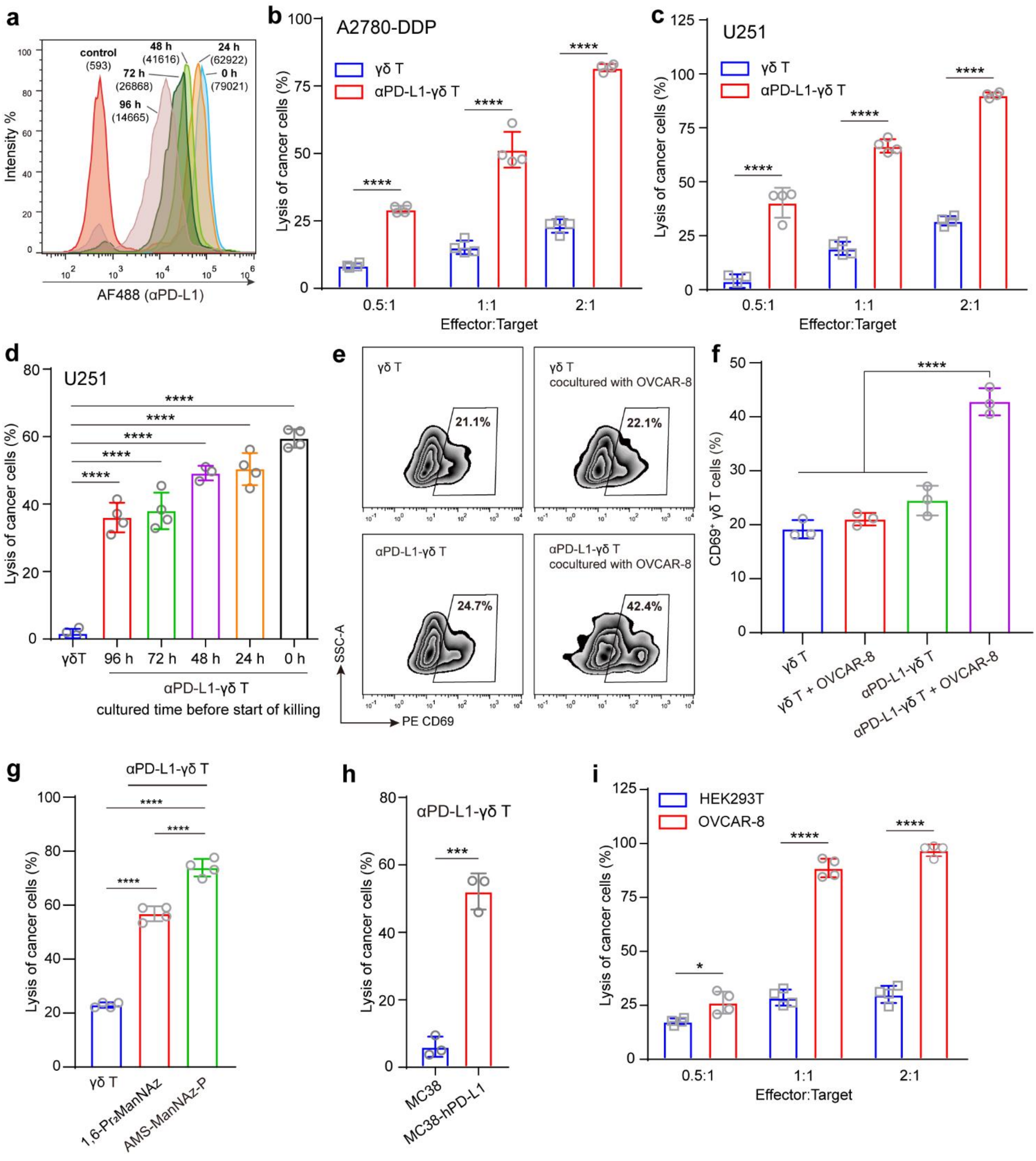

Figure S5. Detailed characterization of αPD-L1-γδ T cells. (a) Representative histograms of flow cytometry analysis on αPD-L1-γδ T cultured for varied durations of time after cell-surface engineering. (b,c) In vitro cytotoxicity of γδ T and αPD-L1-γδ T cells against two PD-L1$^+$ cancer cells A2780-DDP (b) and U251 (c) at different effector: target ratios. (d) Percentages of lysed U251 cells incubated with γδ T cells or αPD-L1-γδ T cells cultured alone for varied durations of time before co-culturing. The effector to target cell ratio was 1:1. (e,f) Representative histograms (e) and bar graph (f) of flow cytometry analysis of γδT cells and αPD-L1-γδ T cells, which were co-cultured with OVCAR-8 cells for 3 h, followed by immunostaining of CD69. (g) Quantification of lysed

OVCAR-8 cells upon incubation with αPD-L1-γδ T cells prepared with 200 μM 1,6-Pr_2ManNAz or 100 μM AMS-ManNAz-P, or γδ T cells. (h) Percentages of lysed MC38 cells and MC38 with overexpressed PD-L1 incubated with αPD-L1-γδ T cells for 24 h. (i) Percentages of lysed HEK293T cells and OVCAR-8 cells incubated with αPD-L1-γδ T cells at varied effector to target cell ratios. In b-d and f-i, the data are presented as mean ± SD (n=3 for f,h and 4 for b-d,g,i). *$p < 0.05$, ***$p < 0.001$, ****$p < 0.0001$ (unpaired Student's *t*-test in h and one-way ANOVA in the rest).

Tabel S1. Information of the primary ovarian cancer cells

ID of primary cancer cells	Origin of tumor cells
Primary #1	Ascites
Primary #2	Ascites
Primary #3	Ascites
Primary #4	Ascites
Primary #5	Ascites
Primary #6	Tumor tissue from ovary

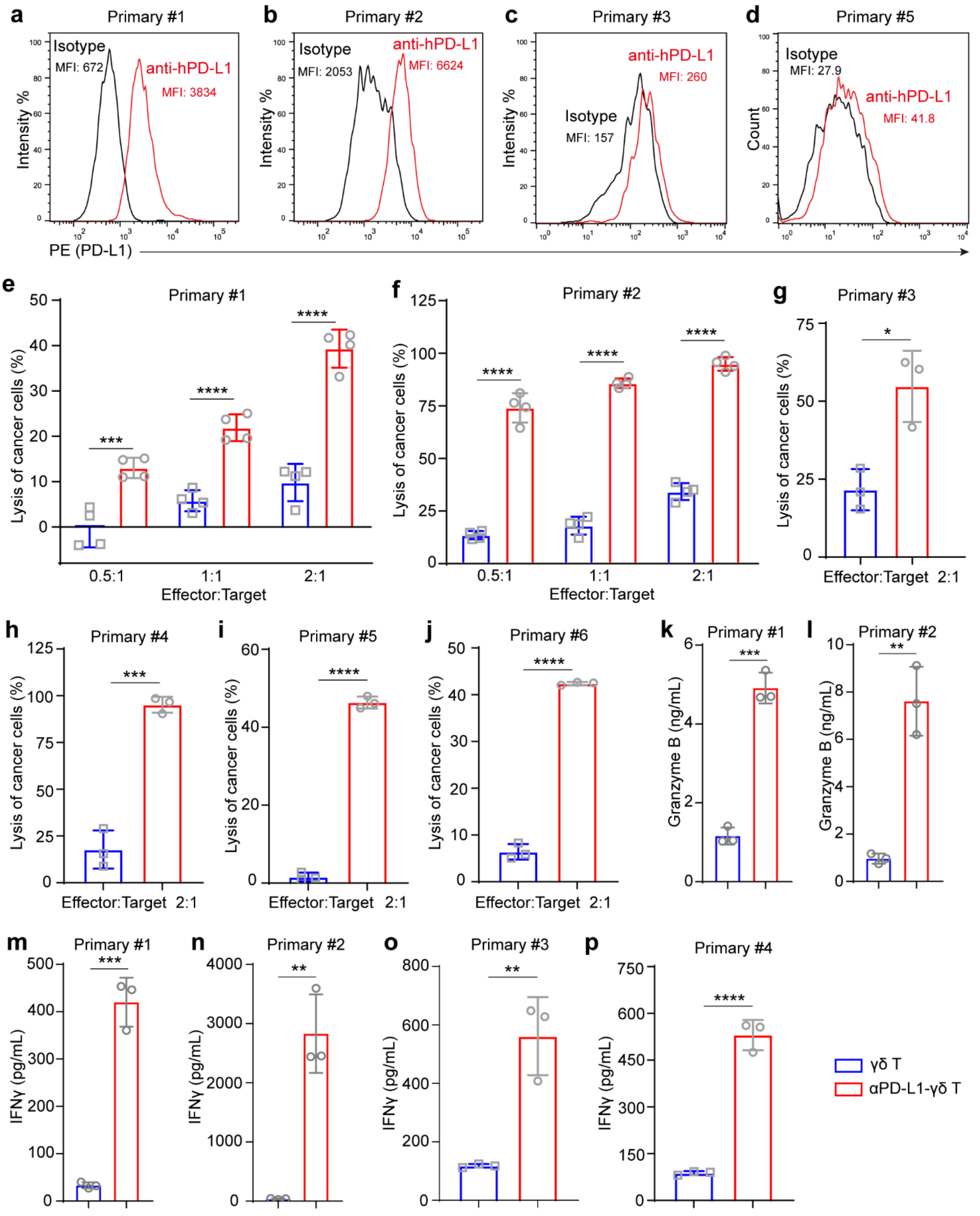

Figure S6. Cytotoxicity of αPD-L1-γδ T cells against patients-derived primary ovarian cancer cells. (a-d) Flow cytometry analysis of PD-L1 expression in primary cancer cells from four patients. (e-j) Killing efficiency of primary cancer cells from patient #1 to patient #6 by γδ T cells and αPD-L1-γδ T cells at varied effector-to-target cell ratios. (k-p) Quantitative analysis of GrB (k and l) and IFNγ (m-p) release from γδ T and αPD-L1-γδ T co-cultured with primary cancer cells at effector-to-target of 2:1. All the data were presented as mean ± SD. n=3 (g-p) or 4 (e,f). * $p < 0.05$, ** $p < 0.01$, *** $p < 0.001$, **** $p < 0.0001$ (one-way ANOVA in e,f and unpaired Student's *t*-test in the rest).

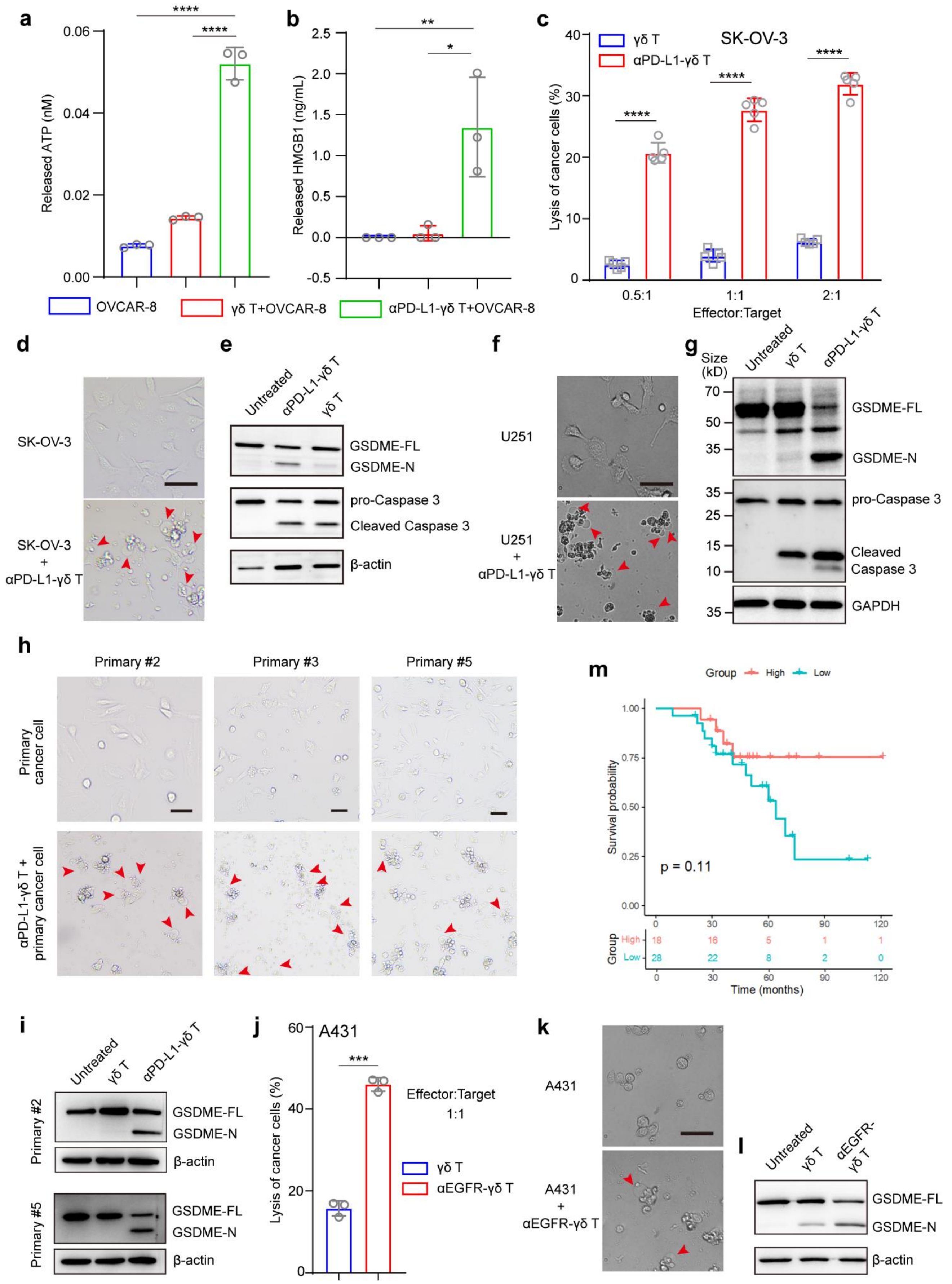

Figure S7. Pyroptosis of cancer cells induced by antibody-γδ T cell conjugates. (a,b) Quantitative

analysis of released ATP (a) and HMGB1 (b) from OVCAR-8 cells incubated with vehicle, γδ T cells, or αPD-L1-γδ T cells. (c) In vitro cytotoxicity of γδ T cells and αPD-L1-γδ T cells towards PD-L1^{+} SK-OV-3 cells under different effector-to-target ratios in 3 h. (d) Bright-field microscopy images of SK-OV-3 cells treated with vehicle or αPD-L1-γδ T cells. (e) Western blot analysis of SK-OV-3 cells treated with vehicle, γδ T cells, or αPD-L1-γδ T cells. (f) Bright-field microscopy images of U251 cells treated with vehicle or αPD-L1-γδ T cells. (g) Western blot analysis of U251 cells treated with vehicle, γδ T cells, or αPD-L1-γδ T cells. (h) Bright-field microscopy images of three primary ovarian cancer cells treated with vehicle or αPD-L1-γδ T cells. (i) Western blot analysis on primary ovarian cancer cells treated with vehicle, γδ T cells, or αPD-L1-γδ T cells. (j) Quantification of lysed A431 cells co-cultured with γδ T cells or αEGFR-γδT cells. (k) Bright-field microscopy images of A431 cells treated with vehicle or αEGFR-L1-γδ T cells. (l) Western blot analysis on of A431 cells treated with vehicle, γδ T cells, or αEGFR-L1-γδ T cells. In a, b, c, and j, the data are presented as mean ± SD (n=3 for a,b,j and 5 for c). *$p < 0.05$, **$p < 0.01$, ***$p < 0.001$, ****$p < 0.0001$ (one-way ANOVA in a-c and unpaired Student's *t*-test in j). In d, f, h, and k, the red arrows indicate the bubble-like structures. scale bars, 20 μm. In e, g, i, and l, GSDME-FL, full length GSDME; GSDME-N, the N-terminal domain of GSDME. The anti-GAPDH or anti-β-actin blots demonstrate comparable loading. (m) Correlation of γδ T cell infiltration with patients' survival probability in ovarian cancer. Data source: GSE32062. High: high γδ T infiltration; Low: low γδ T infiltration. Although the p-value was 0.11, positive correlation between survival and γδ T infiltration could be possibly identified.

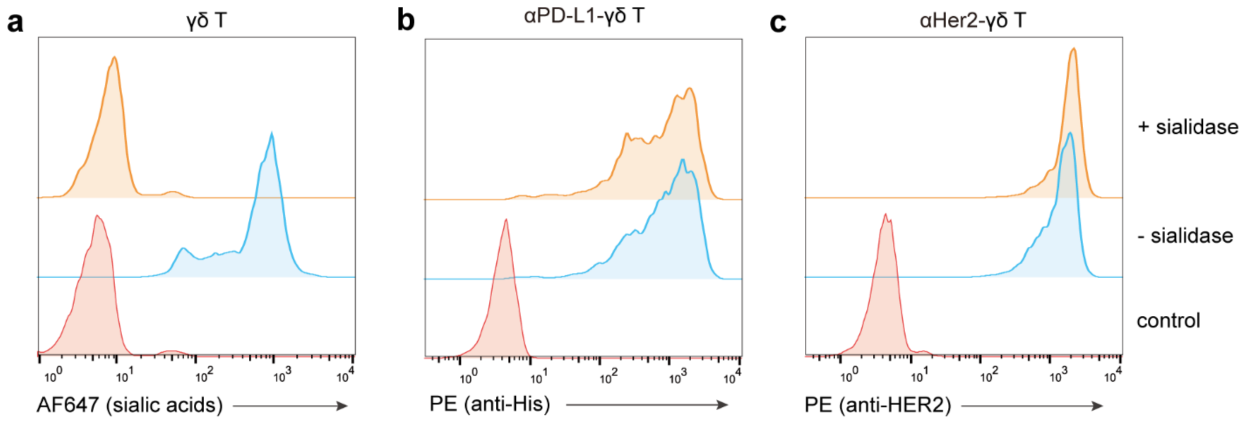

Figure S8. Sialidase treatment of γδ T cells and antibody-γδ T cell conjugates. (a-c) Flow cytometry analysis showing γδ T cells (a), αPD-L1-γδ T cells, and αHER2-γδ T cells treated with vehicle or sialidase. The sialic acids on γδ T cells were stained with tetra-GRABs-BF647, αPD-L1 with PE conjugated anti-His Tag Antibody, and αHER2 with PE conjugated anti-human IgG Fc Antibody.

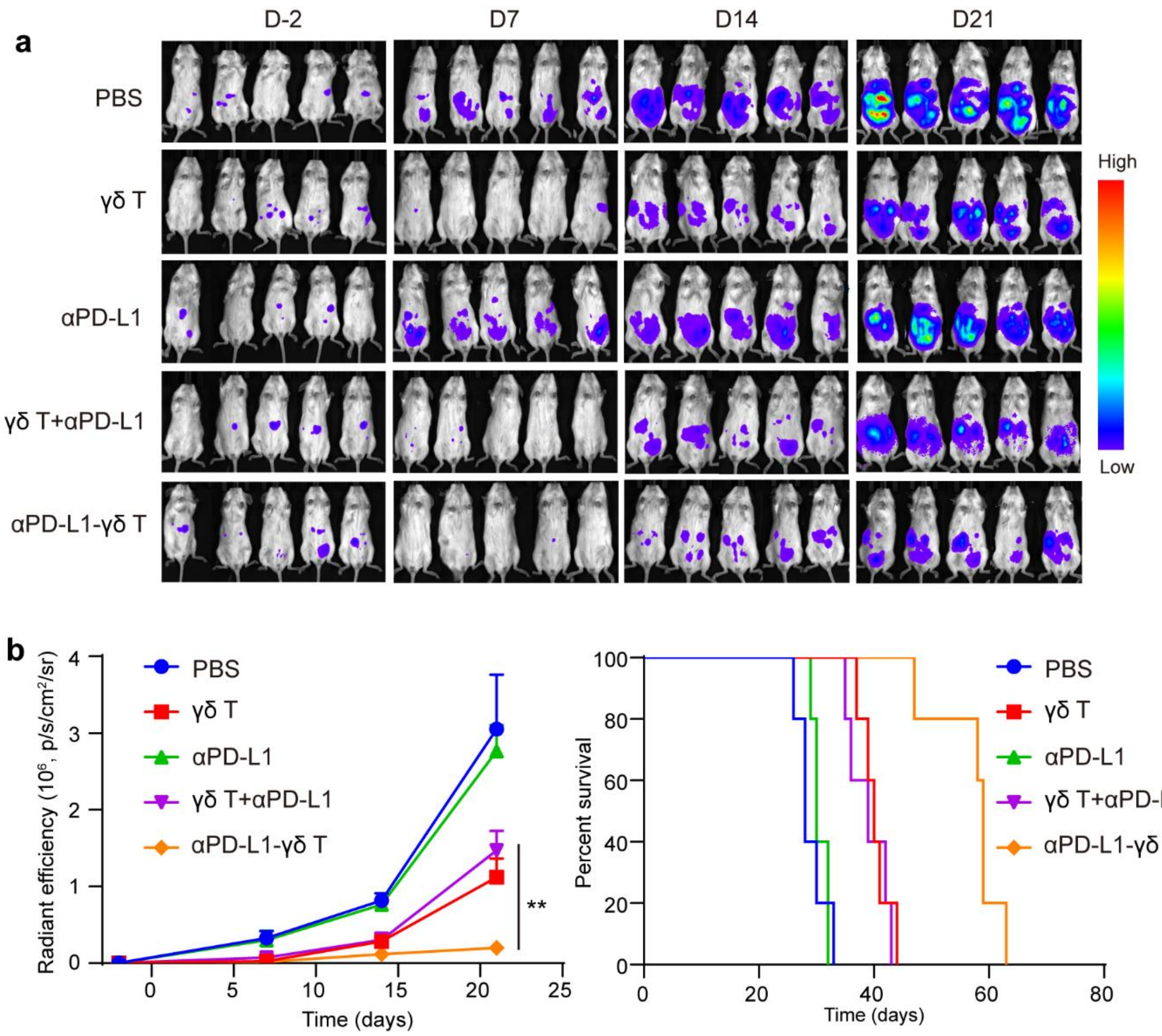

Figure S9. In vivo anti-tumor activity of αPD-L1-γδ T cells compared to the treatment controls. (a,b,c) Time-course BLI images, quantitative analysis of the bioluminescence (b) and survival curves (c) of the tumor-bearing mice treated with vehicle, γδ T cells, αPD-L1, mixture of γδ T cells and αPD-L1, or αPD-L1-γδ T cells compared to the treatment controls. Mice were treated intraperitoneally. n=5 mice per group. In b, data are presented as mean ± SEM. **p < 0.01 (unpaired Student's *t*-test). In c, **p < 0.01 [Log-rank (Mantel-Cox) test].

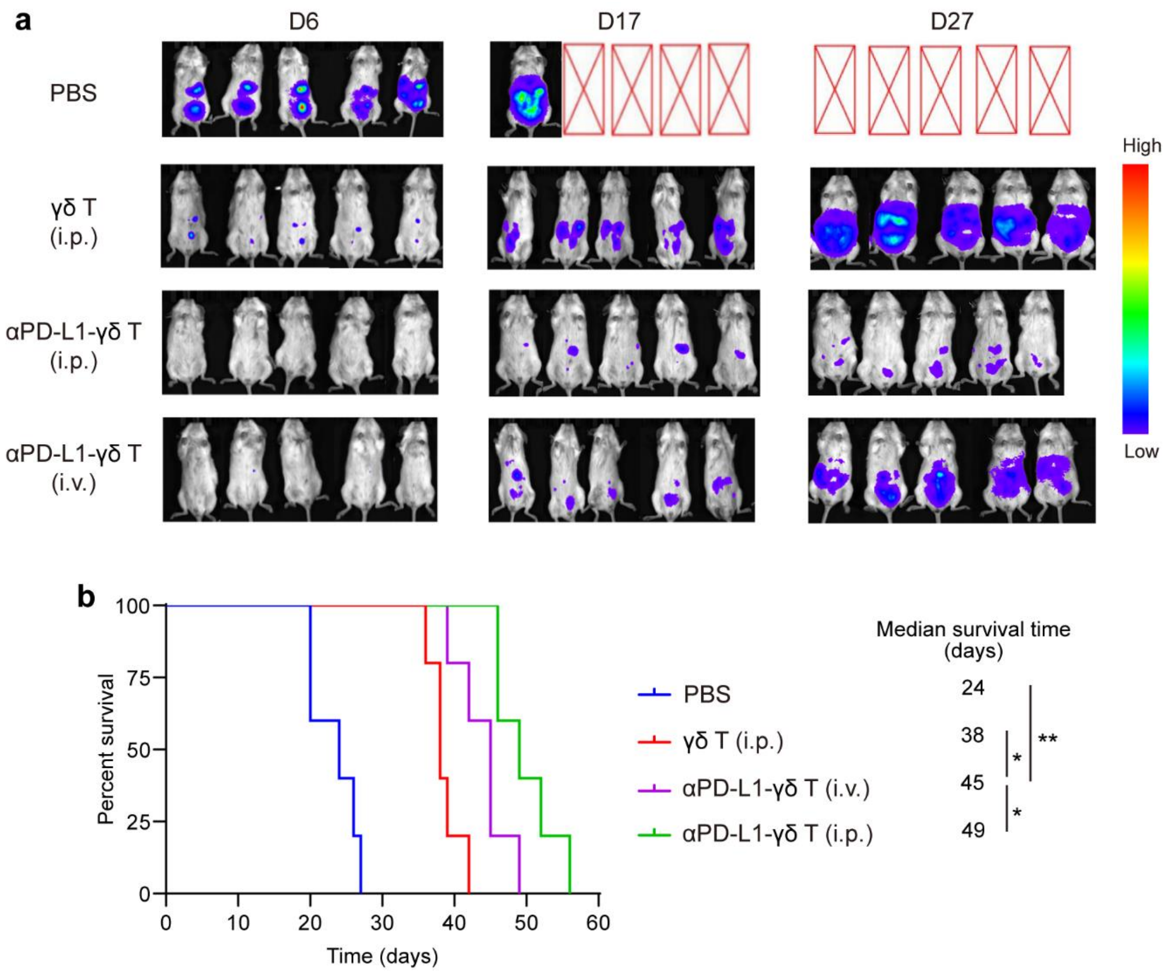

Figure S10. Comparison of the in vivo anti-tumor activity between the intravenously and intraperitoneally administered αPD-L1-γδ T cells. (a,b) Time-course BLI images (a) and survival curves and the median survival times (b) of the tumor-bearing mice treated with vehicle, γδ T cells (i.p.), αPD-L1-γδ T cells (i.p.), or αPD-L1-γδ T cells (i.v.). n=5 mice per group. In b, *p < 0.05, **p < 0.01 [Log-rank (Mantel-Cox) test].

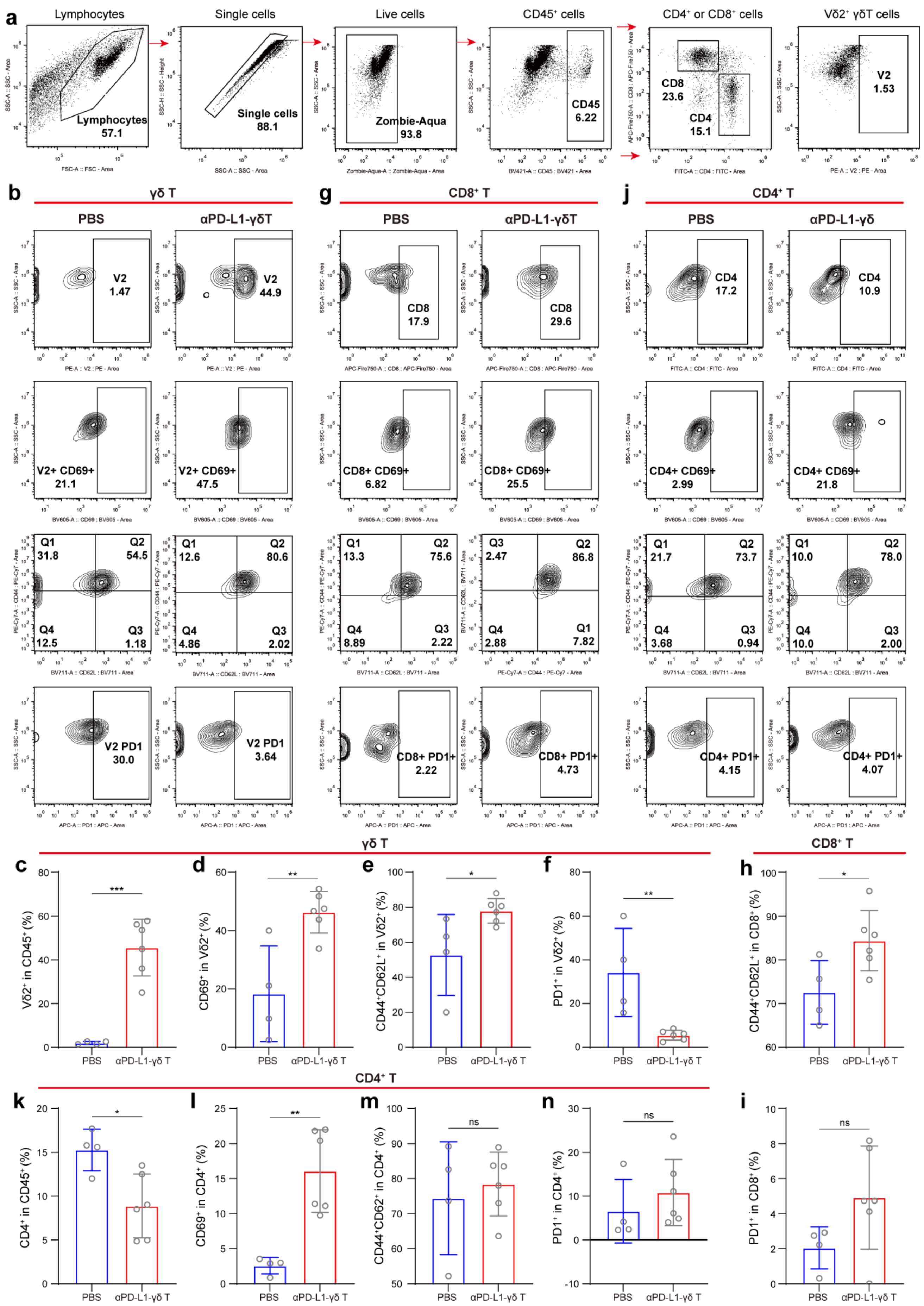

a
Lymphocytes
Single cells
Live cells
CD45⁺ cells
CD4⁺ or CD8⁺ cells
Vδ2⁺ γδT cells
Lymphocytes 57.1
Single cells 88.1
Zombie-Aqua 93.8
CD45 6.22
CD8 23.6
CD4 15.1
V2 1.53
FSC-A :: FSC - Area
SSC-A :: SSC - Area
Zombie-Aqua-A :: Zombie-Aqua - Area
BV421-A :: CD45 : BV421 - Area
FITC-A :: CD4 : FITC - Area
PE-A :: V2 : PE - Area
b
γδ T
PBS
αPD-L1-γδT
V2 1.47
V2 44.9
V2+ CD69+ 21.1
V2+ CD69+ 47.5
Q1 31.8
Q2 54.5
Q4 12.5
Q3 1.18
Q1 12.6
Q2 80.6
Q4 4.86
Q3 2.02
V2 PD1 30.0
V2 PD1 3.64
g
CD8⁺ T
PBS
αPD-L1-γδT
CD8 17.9
CD8 29.6
CD8+ CD69+ 6.82
CD8+ CD69+ 25.5
Q1 13.3
Q2 75.6
Q4 8.89
Q3 2.22
Q3 2.47
Q2 86.8
Q4 2.88
Q1 7.82
CD8+ PD1+ 2.22
CD8+ PD1+ 4.73
j
CD4⁺ T
PBS
αPD-L1-γδ
CD4 17.2
CD4 10.9
CD4+ CD69+ 2.99
CD4+ CD69+ 21.8
Q1 21.7
Q2 73.7
Q4 3.68
Q3 0.94
Q1 10.0
Q2 78.0
Q4 10.0
Q3 2.00
CD4+ PD1+ 4.15
CD4+ PD1+ 4.07
γδ T
CD8⁺ T
c
Vδ2⁺ in CD45⁺ (%)
***
d
CD69⁺ in Vδ2⁺(%)
**
e
CD44⁺CD62L⁺ in Vδ2⁺ (%)
*
f
PD1⁺ in Vδ2⁺ (%)
**
h
CD44⁺CD62L⁺ in CD8⁺ (%)
*
CD4⁺ T
k
CD4⁺ in CD45⁺ (%)
*
l
CD69⁺ in CD4⁺ (%)
**
m
CD44⁺CD62⁺ in CD4⁺ (%)
ns
n
PD1⁺ in CD4⁺ (%)
ns
i
PD1⁺ in CD8⁺ (%)
ns
PBS
αPD-L1-γδ T

Figure S11. Analysis of TILs (a) Gating of various subpopulations of tumor infiltrating lymphocytes. (b) Representative flow cytometry histograms for tumor infiltrating γδ T cells. (c) Percentage of tumor infiltrating $V\delta2^+$ γδ T cells among $CD45^+$ cells. (d) Percentage of tumor infiltrating $V\delta2^+$ γδ T cells with the activation phenotype ($CD69^+$). (e) Percentage of tumor infiltrating $V\delta2^+$ γδ T cells with the central memory phenotype ($CD44^+CD62L^+$). (f) Percentage of tumor infiltrating $V\delta2^+$ γδ T cells with PD-1 expression. (g) Representative flow cytometry histograms for tumor infiltrating $CD8^+$ T cells. (h) Percentage of tumor infiltrating $CD8^+$ T cells with the central memory phenotype ($CD44^+CD62L^+$). (i) Percentage of tumor infiltrating $CD8^+$ T cells with PD-1 expression. (j) Representative flow cytometry histograms for tumor infiltrating $CD4^+$ T cells. (k) Percentage of tumor infiltrating $CD4^+$ T cells among $CD45^+$ cells. (l) Percentage of tumor infiltrating $CD4^+$ T cells with the activation phenotype ($CD69^+$). (m) Percentage of tumor infiltrating $CD4^+$ T cells with the central memory phenotype ($CD44^+CD62L^+$). (n) Percentage of tumor infiltrating $CD4^+$ T cells with PD-1 expression. In c-f, h, i, k-n, the data are presented as mean ± SD (n=4 mice for PBS group, n=6 mice for γδT-αPD-L1 treatment group). ns, not significant, $*p < 0.05$, $**p < 0.01$, $***p < 0.001$ (unpaired Student's *t*-test).

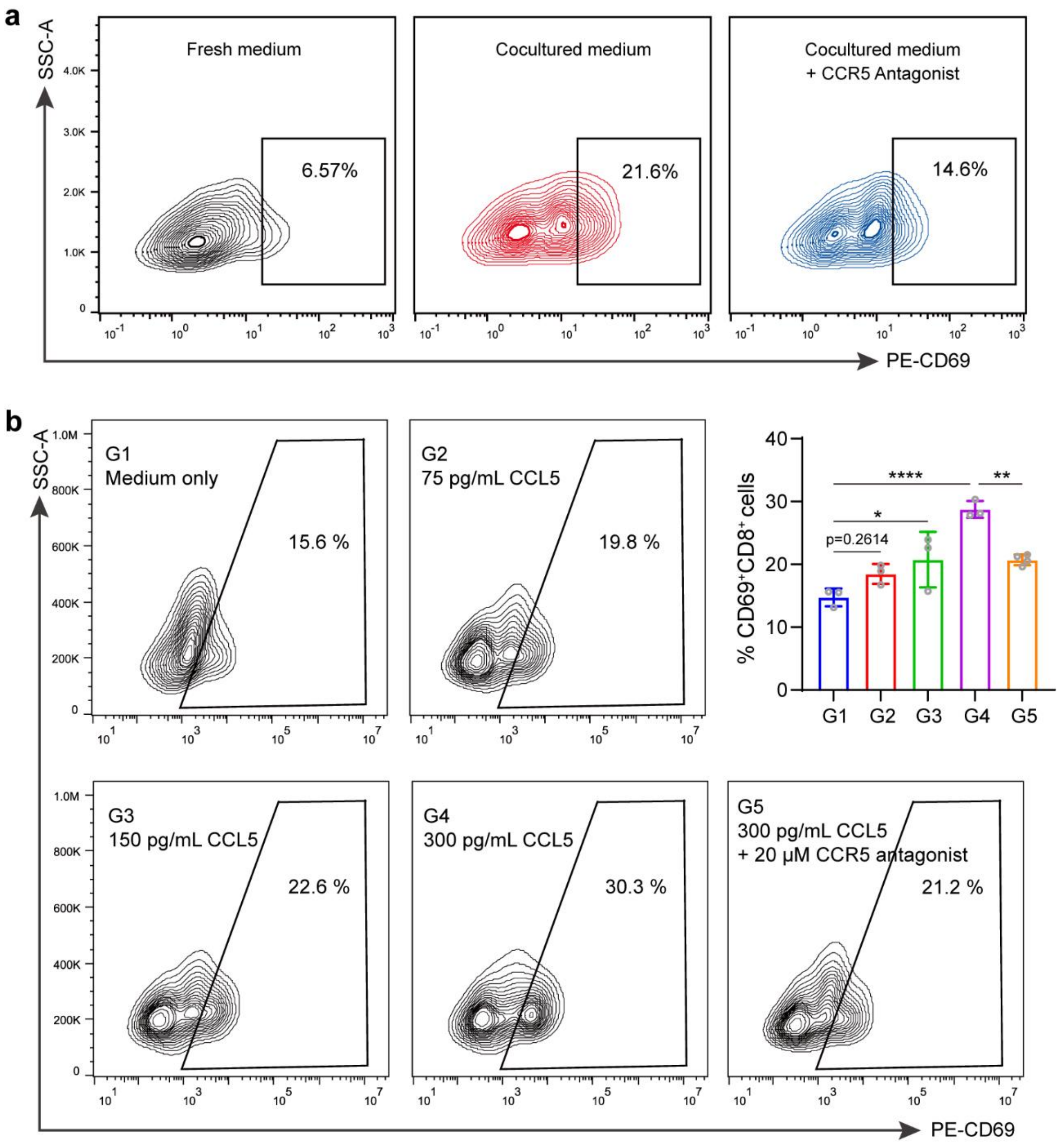

Figure S12. Activation of CD8$^+$ T cells by culture medium and CCL5. (a) Representative flow cytometry analysis of the activation of CD8$^+$ T cells incubated with fresh medium, medium of coculture of αPD-L1-γδ T cells and OVCAR-8 cells, or medium of coculture with CCR5 antagonist. (b) Representative flow cytometry analysis and quantification of the activated CD8$^+$ T cells incubated with CCL5 at varied concentrations or together with CCR5 antagonist. In the bar graph, the data are presented as mean ± SD (n=3 for G1-G4) and 4 for G5). *$p < 0.05$, **$p < 0.05$, ****$p < 0.0001$ (one-way ANOVA).

References

1. Zhang H, Han Y, Yang Y et al. Covalently engineered nanobody chimeras for targeted membrane protein degradation. *J Am Chem Soc* 2021. 143. 16377-82. 10.1021/jacs.1c08521
2. Liu J, Cheng B, Fan X et al. Click-iG: simultaneous enrichment and profiling of intact N-linked, O-GalNAc, and O-GlcNAcylated glycopeptides. *Angew Chem Int Ed Engl* 2023. 62. e202303410. 10.1002/anie.202303410
3. Zeng WF, Cao WQ, Liu MQ et al. Precise, fast and comprehensive analysis of intact glycopeptides and modified glycans with pGlyco3. *Nat Methods* 2021. 18. 1515-23. 10.1038/s41592-021-01306-0
4. Hao Y, Fan XQ, Shi YJ et al. Next-generation unnatural monosaccharides reveal that ESRRB O-GlcNAcylation regulates pluripotency of mouse embryonic stem cells. *Nat Commun* 2019. 10. 4065. 10.1038/s41467-019-11942-y
5. Liang SY, Tang Q, Guo XZ et al. Mutant glycosidases for labeling sialoglycans with high specificity and affinity. *Nat Commun* 2025. 16. 10.1038/s41467-025-56629-9
